# Supplementary material for: Land tenure drives Brazil’s deforestation rates across socio-environmental contexts
Source: Nat Commun. 2022 Oct 1;13:5759. doi: 10.1038/s41467-022-33398-3 (PMC9526711; doi:10.1038/s41467-022-33398-3)
Supplement: Supplementary file 1 — Supplementary information [file 41467_2022_33398_MOESM1_ESM.docx]

**Supplementary information for:**

**Land tenure drives Brazil's deforestation rates across socio-environmental contexts**

*Corresponding authors: andrea.pacheco@ilr.uni-bonn.de, carsten.meyer@idiv.de

**This appendix includes:**

Supplementary Methods

Supplementary Figures 1-5

Supplementary Tables 1-8

Supplementary References

Supplementary Methods

**1. Study context: land tenure in Brazil**

Modern land-tenure regimes as they exist in Brazil today – with all rights and regulations that apply to them – exemplify the complex historical processes of land distribution common to tropical nations. Deliberate colonization of the central and northern regions was encouraged since the 1930s, but occurred at a large scale during the period of military dictatorship (1964-1985). The Land Statute enacted in 1964 brought forth the concept of land fulfilling a ‘social function’ – creating legal instruments for land expropriation and taxation as official means of land redistribution and regularization. In parallel, the Forest Code created in 1965 (Federal Law No. 4.771) required private landowners to leave 20-80% of the land under native vegetation, depending on the region. Soon thereafter, in the 1970s, The National Institute of Colonization and Agrarian Reform (INCRA) was created with the purpose of reclaiming unproductive land and settling the landless. Settlers were specifically incentivized to replace forest with cattle pastures or croplands. However, the official creation of these settlements was largely ineffective and many were never formalized – oftentimes large ‘unproductive’ farms persisted, and illegal occupation of lands continued to be common. At the same time, in addition to the existing occupants of these regions (e.g., indigenous peoples, rubber *Seringueiros,* and riverine communities), land grabbers staked claims on land by counterfeiting land titles (*Grilheiros)* or creating ‘ghost’ property owners ^1–3^.

When the dictatorship ended and a new constitution was written in 1988, protected areas (PAs) were planned on existing public lands, and the law recognized autonomous land rights for indigenous peoples and quilombolas for the first time. Still, the formalization of many these areas took 10 years to even begin, with registration and demarcations processes still ongoing to date. On the other hand, land-use rights and (dis)incentives for deforestation in public and private lands were targeted through a variety of environmental policies and programs. This included efforts specifically focusing on mitigating deforestation in the Amazon and the Cerrado biomes, often incorporating issues relating to land tenure regularization (e.g. PPCDAm (2004), PPCerrado (2010), REDD+, and the soy moratoria (2006)) ^4,5^. It further included the regularization of *de facto* public and private lands resulting from the colonization process of the 1970s as part of the new Forest Code – the Native Vegetation Protection Law (Lei 12.651 2012). The new Forest Code provides incentives for the voluntary registration of rural public and private properties in the official Rural Environmental Cadastre (CAR), facilitating GIS-based forest monitoring of tenants’ compliance with requirements to maintain certain levels of native vegetation coverage (20-80% depending on the biome ^6,7^). Altogether, these regulations, policies, and programs have roughly defined the *de jure* and *de facto* tenure regimes in Brazil for the past 50 years (Supplementary Table 2).

**2. Data**

**2.1. Land tenure data**

We used the publicly available data on land tenure compiled by Imaflora (v. 1812) ^8^. This spatially explicit parcel-level dataset maps land-tenure for 83.4% of the Brazilian territory. It is based on 18 official, most up-to-date data sources, which were integrated using an expert-vetted system to systematically resolve data conflicts resulting from, e.g., overlapping land claims due to due illegally fabricated land titles and/or mapping errors ^9^. These data likely represent the most reliable and comprehensive parcel-level land-tenure information available for any large tropical country. Nevertheless, we acknowledge remaining uncertainties in the depicted spatial patterns, particularly in certain regions where overlapping land claims are reportedly higher than elsewhere (*9*). Our analytical approach across multiple regions (see sections 3.2 and 3.5) partly buffers against possible biases introduced from high data uncertainties in any particular region.

For most tenure categories, the available data lack, or have incomplete information on the date of each parcel’s formalization (i.e., titling or demarcation). Despite possible changes in official ownership status, it can be assumed that for the majority of parcels, the basic type of tenancy (e.g., public institutions vs. indigenous communities vs. private individuals) did not change over the course of our study period. However, as we deemed this assumption problematic for certain tenure categories, we took several steps to minimize possible bias in our statistical analyses and conclusions. Firstly, we performed all analyses over multiple spatial and temporal extents and assessed whether results for Brazilian subregions and time periods with known changes in tenure patterns were qualitatively consistent with those for ‘tenure-stable’ regions/periods. Secondly, we excluded tenure sub-categories defined via programs that only came into existence after our study periods began. Thirdly, we performed robustness tests for selected tenure categories with documented ‘treatment’ dates, where we filtered out parcels for which today’s tenure category was non-existent or unclear at the beginning of the respective study period. Fourthly, we assessed possible biases in our quasi-experimental setup due to remaining statistical imbalance, omitted variables, and systematic differences in initial forest cover between ‘treatment’ and ‘control’ units. We outline the specific steps taken in our description of the tenure categories analyzed (see section 2.2) and of our study design and statistical approach (see 3.3-3.6).

**2.2. Categorization of land-tenure regimes**

Many countries employ unique categories or subdivisions of land-tenure forms, which makes international comparisons difficult. For instance, the Imaflora dataset distinguishes 14 different tenure categories, including several different subcategories of private and public lands that are products of Brazil’s specific land-administration history. However, a central aim of this study was to identify land tenure effects that might be transferable across different contexts (i.e., potentially including non-Brazilian regions). Therefore, we lumped several Brazil-specific tenure categories to more closely correspond to classical types of land-tenure regimes that are also present in other tropical forest nations, while still sufficiently specific to the context of Brazil to also enable country-specific conclusions. The distinguished tenure regimes are characterized by specific ‘bundles of rights’ ^10^ and responsibilities that regulate how the tenants can interact with their land resources (see Supplementary Table 2).

*Private lands (hereafter ‘private’)*. This category includes lands that are privately owned by individual persons, companies, or other entities (but not communities; see below). Of all tenure regimes, private tenure guarantees tenants the most extensive set of rights (Supplementary Table 2), although some resource-withdrawal rights are regulated through existing agricultural and environmental policies. We combined private properties from different sources (CAR, SIGEF) under this category. While a small percentage of these private lands may have shifted tenure categories during our study period, most had already been settled and formally recognized as private lands before the mid-1980s (e.g. ^11^; note that subsequent changes in the specific property owners are not relevant to our study). By contrast, we excluded all private properties titled under the Terra Legal program from our analyses, as this program only started in 2009 and, accordingly, these properties experienced shifts in tenure categories during our study period. Note that deforestation effects of property titling under the Terra Legal program were recently the focus of different study ^12^.

*Undesignated and untitled public lands with poorly defined tenure rights (hereafter ‘undesignated/ untitled’)*. Common to all lands included in this category is that while they are publicly owned, the state has not formally assigned them to any purpose, or, if they are occupied by settlers, has not recognized any tenure rights of them (e.g., via registration or titling). Withdrawal use rights on undesignated/untitled lands are usually not regulated, and *de jure* existing regulations are typically not enforced. Where rural settlements were historically permitted, settlers were required to put at least 80% of the occupied land area to ‘productive use’. Unlike private landowners, however, these settlers never had any exclusion rights, alienation rights, or rights to due process (neither formally nor otherwise guaranteed; Supplementary Table 2). We merged public properties listed in the Imaflora dataset as either ‘undesignated lands’ or ‘rural settlements’ into this category, but excluded all rural-settlement parcels that are part of the Terra Legal program. Our reason for this exclusion was that the specific design of this program may have incentivized some settlers to clear forestland in anticipation of the later titling process ^12^, which could have biased our perception of the normal effects of untitled/undesignated regimes on forests. Untitled/undesignated lands today have had this status throughout the 1985-2018 period.

*Conservation-focused tenure regimes*. We followed the classification of conservation-focused tenure regimes used by the Ministry of Environment of Brazil, corresponding to the commonly distinguished categories of fully protected areas (*Unidades de Conservação de Protecão Integral*) and sustainable-use areas (*Unidades de Conservação de Uso Sustentavel*). These two categories mainly differ in their access and withdrawal-use rights, with strict-protection regimes severely restricting access and prohibiting all extraction or withdrawal, whereas sustainable-use regimes afford certain access and withdrawal rights, as long the long-term sustainability of natural resources is ensured (Supplementary Table 2). Neither category affords alienation rights to the citizenry or communities that are technically the main rights holders. Unlike the private and undesignated/untitled lands included in our study, substantial percentages of the parcels under either conservation-focused tenure regime have only come under the respective regime during the course of our study period. Beyond qualitatively assessing consistency of results between more and less ‘tenure-stable’ regions and periods, we thus performed additional robust tests for these categories. Specifically, we repeated our statistical analyses on time-filtered datasets that excluded parcels that either were not under today’s tenure category for at least the latter 80% of the respective study period or for which the formal designation date was unknown, using establishment dates from ^13^. Note, Areas of Environmental Protection (*Áreas de Proteção Ambiental*) are excluded from the Imaflora dataset, and thus not included in this analysis ^8^.

*Indigenous peoples and local community (IPLC) based tenure regimes*. Brazil distinguishes three main categories of community-based tenure – indigenous, quilombola, and communal. To analyze hypothesized effects of IPLC tenure, we decided to maintain the distinction between these three tenure regimes, due to their very different histories, legal statuses, and granted bundles of rights (Supplementary Table 2). Specifically, indigenous lands are statutorily publicly owned, but managed by indigenous communities with ancestral claims, who are granted strictly non-commercial withdrawal (i.e., subsistence-use) rights. We combined both homologated (formally recognized) and non-homologated indigenous lands into a single category, as this distinction mostly reflects differences in *de jure* formalization, rather than in the tenure rights *de facto* assumed on the ground. Quilombola lands, by contrast, are communally managed yet privately owned by self-defined communities of descendants from escaped African slaves. Many quilombos have been granted official titles, which legally guarantee commercial as well as non-commercial withdrawal rights. However, quilombola as well as indigenous communities do not have alienation rights (i.e., their lands cannot be sold, leased, used as business collateral, or dismembered).

The third type of IPLC lands, communal lands (*Territórios Comunitários*), are publicly owned but grant certain rights to different groups of self-defined communities traditionally managing forest resources (e.g., *Castanheiros*, *Seringueiros*). Communal tenure regimes are relatively heterogeneous in their rights regulations (Supplementary Table 2). They typically afford the tenants non-commercial withdrawal rights, but the afforded commercial-withdrawal, management, exclusion, and alienation rights vary and are not always clearly defined. Communal tenure is generally the least formalized tenure regime in Brazil, which is also reflected in limited due-process rights (Supplementary Table 2). We decided to restrict our main analyses of IPLC tenure regimes to indigenous and quilombola tenure, both because of the ambiguity of communal lands’ bundles of rights and because there were insufficient registered communal land parcels to support our quasi-experimental design in all biomes except Amazonia. However, we additionally provide results for communal tenure in Supplementary Figs. 1-3, and Source Data file.

Many lands claimed by IPLCs are still unmapped or are mapped but not yet officially registered ^1,2^ and were thus excluded from our analyses. Brazil’s indigenous and quilombola communities have long been tenants of their lands, and the recognition of their tenure rights through the 1988 constitution was a result of ongoing political and legal processes that precede our period of analysis (1985-2018). Later formalization steps such as demarcation and registration thus constituted changes from informal to formalized versions of the same *de facto* tenure regimes. In the case of indigenous lands, Law 6.001 of 1973 uses the reference to forest populations in the constitution of 1967 to define indigenous lands as reserved areas occupied by forest populations or indigenous peoples. The law prohibited any activity that would displace occupants of these lands (including buying/selling or renting), and non-indigenous peoples were prohibited from hunting, fishing, or conducting other extractive or agricultural activities on these reserved areas. Furthermore, FUNAI has been part of all demarcation procedures of indigenous lands since 1976, despite constant changes in the specific legal procedures in place. Similarly, quilombola lands have in most cases *de facto* existed throughout the past 100 years, despite varying levels of social conflict and legal recognition. Quilombo activists had formed strong political movements to demand land rights ^14^ after the colonization process of the 1970s brought many settlers to the central, north, and northeastern regions of Brazil, where most quilombola lands are located. Recognition of their specific bundle of rights began in the mid-1980s, coinciding with the first period of our analysis, and culminated in their legal recognition through the 1988 constitution and the establishment of a dedicated institution to demarcate quilombola lands (Fundacão Cultural Palmares). Since then, several legislative documents have further outlined demarcation processes, which INCRA took over in 2009.

We omitted military lands, urban and transport-related lands, and water from our analyses, as these are less relevant to the hypothesized mechanisms relating land-tenure regimes to forest-to-agriculture conversion. We thus focused the main analyses on six categories of land-tenure regimes: undesignated/untitled public, private, fully protected, sustainable use, indigenous, and quilombola.

**2.3. Land-use change data**

We used the 30-m-resolution annual land-cover/use dataset provided by Mapbiomas ^15^ for our calculations of forest-to-agriculture conversions over different time periods between 1985 and 2018. We defined forest-to-agriculture conversions as any case where either natural or plantation forest cover, savanna, or mangrove cover changed to any category of farming (pasture, agriculture, annual, perennial, and semi-perennial crops, and mosaic of agriculture and pasture) over the respective time period considered.

**2.4. Covariate data**

We used a set of covariates known to influence forest-to-agriculture conversion which are have been shown to be relevant for policy-makers when deciding on shifts in tenure regimes under many different contexts. These include market accessibility (represented by travel time to nearest city; ^16^), agricultural suitability (represented by slope and elevation; ^17^), population density ^18^, and parcel area in ha ^8^. See sections 3.3 and 3.6 for details on covariate use. Agricultural suitability and accessibility have been shown to be key determinants of achievable land rents, and are thus good proxies for the opportunity costs associated with ‘assigning’ land to any given tenure regime. These covariates thus also reflect general tendency of potentially more profitable (i.e., private) tenure regimes to be established near markets and in agriculturally suitable lowlands, whereas likelihoods of acknowledging IPLC rights or creating conservation regimes are higher in more remote and/or steeper terrains of relatively lower economic importance ^9,19–23^. Higher population density can translate into higher pressure on policy-makers to ‘assign’ or recognize tenure regimes that allow the use of natural resources (e.g. private, IPLC), whereas lower population density implies lower economic, social, and/or political costs of creating conservation regimes or of leaving lands undesignated. Finally, we included parcel area in ha ^8^ because the price landholders pay for receiving land titles depends on a property’s size ^12^ and because certain forest/agricultural policies apply differently to certain tenure regimes depending on parcel size (e.g., requirements for private landholders to maintain a certain percentage of forest cover, or requirements for rural settlements to maintain certain levels of agricultural productivity). Furthermore, compliance with these policies has also been shown to depend on property size ^24^.

**3. Study design and analysis**

**3.1. Overview**

Our goal was to assess and synthesize the direction, strength, and generality of the longer-term effects that shifts between alternative land-tenure regimes typically have on forest-to-agriculture conversion rates. Rather than near-term impacts of specific tenure-intervention events (e.g., titling), we thus wanted to capture the differential impacts of alternative regimes over periods of several years to decades, and assess how consistent and thus, how transferable effects of these regime differences are across different regional-historical contexts.

To estimate causal effects from observational data, we used a quasi-experimental study design that combined matching with a generalization assessment and subsequent regression analysis that incorporates weights to estimate population-wide effects ^25^. To test the extent to which the effects were consistent across regions and periods within Brazil with diverse socio-environmental settings, and thus potentially transferable to other tropical forest regions, we systematically repeated all analyses over 49 different combinations of spatial and temporal extents. We formally synthesized these scale-specific effects via two complementary approaches, designed to assess *i)* the consistency of the net direction of the effects, and *ii)* the consistency of their relative strength in comparison to the effects of other tenure regimes.

**3.1. Plausible changes in land-tenure regimes**

We defined ‘land-tenure regime’ as the combination of tenure-related governance factors that exist over a given parcel of land and are stable over a certain period of time. This includes the bundle of rights associated with the respective tenure category (Supplementary Table 2), but also the implications that these rights may have for tenure security, as well as the tenure categories’ predispositions for being subject to particular types of policies or regulations. Correspondingly, we define ‘tenure-regime change’ as a stable shift from one such regime to another. Tenure-regime changes are thus not instantaneous events, but gradual processes that may involve different legal and administrative acts (e.g., titling, registration, or other steps) and will only be completed after the resulting changes in rights, regulations, and perceptions have come into effect.

We focused on major types of tenure-regime changes corresponding to tenure-intervention processes that are commonly observed across the tropics and are related to different sustainability questions. Firstly, we focused on shifts from undesignated/untitled public regimes with poorly defined tenure rights to private tenure regimes, which over the past decades have been the most common outcomes of tenure interventions (e.g., through formal titling, registration, or other tenure-regularization processes). Secondly, we considered shifts of such undesignated/untitled to conservation-focused tenure regimes, corresponding to designation of public lands as fully protected or sustainable-use areas. Thirdly, we considered shifts from either undesignated/untitled or from private regimes to community-based tenure regimes, corresponding to processes of recognizing tenure rights claimed by IPLCs (which might involve anything from simple registration to multi-year court battles). Finally, we considered changes from different public regimes to private tenure regimes, corresponding to privatization of state-owned lands (which may affect undesignated/untitled, but also conservation, indigenous, or other public lands ^26,27^).

We note that the specific analysis methods we used (see 3.3) do not *per se* restrict the direction in which the estimated effects may be interpreted. As such, an estimated deforestation-increasing effect of replacing a public with a private tenure regime (e.g., through privatization of formerly protected areas) might equally be interpreted as a deforestation-decreasing effect of the same magnitude of a regime change in the opposite direction (e.g., via government seizure and subsequent protection of private lands). Similarly, we could not claim, in these particular tests, that any specific characteristics of either the private nor the public tenure regime would cause the observed difference in deforestation. Instead, we interpret the observed deforestation differences more neutrally as being due to the combination of relevant differences between the two tenure regimes.

**3.2. Analyses at different spatial and temporal scales**

Insights on the environmental implications of land-tenure policies from Brazil are commonly transferred to inform policy strategies in other tropical regions, reflecting the data limitations in most other countries, Brazil’s extensive experience in linking tenure reform with environmental policies, and Brazil’s own active role in South-South development cooperation ^11,28,29^. Notwithstanding this practice, causal effects in complex human-environment systems are often highly context-specific ^30^, which can limit the transferability of conclusions from contextually bound studies. Yet, effects shown to be consistent across very different socio-environmental contexts may also hold in yet other contexts. Based on this tenet, we defined 49 different combinations of spatial and temporal extents of analysis, corresponding to distinct socio-environmental contexts characterized by different bioclimatic regions with distinct agricultural sectors and environmental governance regimes, as well as by different historical time periods that saw different policies, macro-economic events, and trends in deforestation. We repeated the full statistical analysis procedures for each tenure-regime comparison for each of these spatiotemporal scales (see below).

We defined a ‘large’ spatiotemporal extent covering the entire spatial extent of Brazil and capturing the net agriculture-to-forest conversion over the full 1985-2018 period. In addition, we ran all analyses over the same temporal extent but over the six narrower spatial extents defined by Brazil’s biomes (Amazônia, Caatinga, Cerrado, Mata Atlântica, Pampa, and Pantanal). These biomes correspond to highly distinctive environmental and socioeconomic conditions, ranging from early-colonized, economically diversified, and intensively governed regions, to newly emerging agroeconomic frontiers, economically marginalized drylands, and remote rainforest areas. Additionally, we ran all analyses over both large and narrower spatial extents over six narrower temporal extents, which we defined to coincide with major deforestation periods in Brazil. The first temporal extent (1985-1990), during which several tenure types first received legal recognition, was a time of deep economic crisis, high inflation rates, and high levels of social unrest. The period of 1990-1995 represents a time of economic recovery; elections in 1994 contributed towards increasing access to agricultural credit in several key federal states, agricultural mechanization increased in key regions, and El Niño-related droughts and fires added to a sharp peak in deforestation rates in 1995. During 1996-1999, as well as 2000-2004, there was steady economic growth, with deforestation peaking again in 2004. 2005-2012 marks a period of declining deforestation rates after a drop in global soy prices and renewed environmental legislation and enforcement focused on the private sector (e.g., the soy moratorium of 2006; ^31^, the proposal of REDD+; ^32^). Finally, the period of 2013-2018 corresponds to the most recent amendment of the Forest Code, which has been widely criticized for its leniency in granting amnesty for past deforestation and lowering the requirements for restoration ^6^.

**3.3. Creating quasi-experiments on shifts in land-tenure regimes**

To be able to estimate causal effects from observational data, we used a quasi-experimental study design, and combined matching with a subsequent regression analysis that included weights to generalize from matched samples to population-wide effects ^25,33^. Matching addressed the bias that would arise due to ‘treatment’ assignment not being independent of the outcome. For instance, landscapes (e.g. savannas) may be more prone to certain land-uses (e.g. agriculture), which may influence ‘treatment’ assignment (e.g. titling agricultural land to a private land holder, recognizing a forest as part of an indigenous land claim). If simpler regression designs were applied to the tenure dataset due to this non-random assignment of tenure regimes into experimental ‘treatment/control’ groups, results would be highly biased and model dependent due to high levels of imbalance. Thus, we specifically used coarsened exact matching (CEM; ^25^), which addressed this bias by pruning the dataset to matched pairs of parcels that were highly similar with regard to potentially confounding variables in a stratified way. We conducted one-to-one matching, meaning that each pair of parcels contained one parcel coded as ‘treatment’ under one of two compared alternative tenure regimes, and another (the ‘control’ or ‘counterfactual’) under the respective other regime. Effects were subsequently estimated via regression on the balanced-improved uncoarsened data subset.

We note that other quasi-experimental designs such as difference-in-difference (or before-after-control-impact) are more suitable than matching in certain situations, and are commonly used for estimating near-term effects of specific tenure interventions such as titling ^12^. However, such designs are difficult to apply to processes such as tenure-regime shifts that may only manifest gradually over time through combinations of different events. Moreover, they generally cannot be used where longitudinal datasets of sufficient spatiotemporal scope are not available for all experimental treatment types (as is the case for most land-tenure types across the tropics). Therefore, we believe that cross-sectional comparisons using matched data was currently the most feasible approach for addressing our question. However, we caution that our data do not capture any actual long-term tenure-regime shifts, but merely differences in tenure-regimes among otherwise highly similar parcels. Thus, our estimated effects should be interpreted accordingly, i.e., as the hypothetical effects of fully completing a tenure-regime shift under the assumption that everything else be kept constant.

We also note that our analysis relies on the non-interference assumption, i.e., that the outcome of an observation is not affected by any other ‘treatment’. This would require the deforestation of a land parcel under a particular tenure regime to be unaffected by neighboring (or even distant) tenure regime dynamics. While this would be difficult to prove empirically, recent research on deforestation ‘spillover’ effects of both conservation and indigenous regimes onto other tenure regimes found non-significant or minimal effects for most of Brazil ^34^. This study found only one case of spillover effects in the Amazonian state of Pará during 2000-2004, where conservation regimes were shown to cause decreasing deforestation outside their boundaries, whereas indigenous lands caused ‘leakage’, i.e., increasing deforestation elsewhere. This means that, although we cannot rule out there may be some ‘spillover’ effects at play in our study system, these are likely negligible in most cases. In those cases where these effects might not be negligible, our results would likely underestimate deforestation-decreasing effects of conservation regimes, while overestimating deforestation-decreasing effects of indigenous lands in Amazonia.

Estimating causal effects via matching also requires the assumption that there is no ‘unobservable-variable’ bias due to omitting important confounders. We controlled for five commonly used confounders that are known to influence forest-to-agriculture conversion (see 2.4). We additionally minimized risks of unobservable-variable bias by *i)* including fixed effects for federal states to capture subnational governance differences, *ii)* clustering our standard errors by municipality, and *iii)* assessing sensitivity of our results against potential omitted-variable bias using Rosenbaum bounds (see 3.4 for further details). Moreover, we specifically assessed possible bias due to systematic differences in initial forest cover (see 3.6). We note that causal analyses of instantaneous/short-term events would typically control only for pre-treatment covariates, to avoid the risk that covariates on the causal ‘pathway from exposure to outcome’ might block part of the investigated effect ^35^. However, as we analyzed longer-term effects of alternative stable tenure regimes, our treatments acted continuously throughout the respective study period. Corresponding to such continuous treatment, we averaged the time-variant population-density variable over the years of the respective period (including linearly interpolated/extrapolated values as necessary).

We applied the coarsened-exact matching algorithm implemented in the ‘*cem’* package ^36^ in R versions 3.5.1-4.0.2 ^37^. CEM involves temporarily ‘coarsening’ each confounding variable into bins (predetermined strata). We used automated coarsening for elevation, slope, and human-population change, but manually defined bins for travel time to nearest city and for parcel area. We divided travel time to nearest city into bins of 0-2, >2-6, >6-12, >12-24, and >24 hours, and parcel area into 14 bins of 0-2, >2-5, >5-15, > 15-50, >50-100, >100-500, >500-1,000, >5,000-10,000, >10,000-50,000, >50,000-100,000, >100,000-500,000, >500,000-1,000,000 ha. By conducting CEM individually for each of our defined spatiotemporal extents, we assured exact matching considering the total spatial and temporal variation in the covariates at the respective scale.

While CEM, in particular, has a range of advantages over other matching approaches ^25^, identifying exact matches is generally difficult when there is little overlap in parcel-level similarity among covariates. However, the large number of parcels (~4 million) in the Imaflora dataset allowed us to retain sufficiently large data subsets for unbiased parameter estimation for most tenure-regime comparisons and spatiotemporal scales (44 to 34,218 of unique observations, corresponding to ≥6 observations per parameter; ^38^; see Source Data file). Due to very small numbers of matched parcels (4 to 28), we did not estimate effects for communal tenure regimes in the Caatinga, Cerrado, and Mata Atlântica, nor for any regime other than undesignated/untitled and private in the Pampas and Pantanal biomes.

We use the *L_1_* measure developed by King et al. ^36^ to calculate remaining imbalance post-matching. Across all datasets that we used for our scale- and tenue-regime-specific tests, CEM improved balance by 5-79% (0-73% for time-filtered tests) (Source Data file). Imbalance post-matching ranged from 0.10-0.76, meaning that our datasets achieved between 24% and 90% balance in covariate values. To make cases of high remaining imbalance post-matching easily recognizable, we visualize imbalance as transparency gradients in all plots of estimated effects (Fig. 2, Supplementary Figs. 1-4). Moreover, we explicitly incorporate imbalance into our cross-scale synthesis of results (see 3.5).

**3.4 Improving generalizability and estimating Average Treatment Effects (ATE)**

In this study we defined our estimand of interest as Average Treatment Effects (ATE), i.e. the average difference between two tenure regimes (a ‘treatment’ and ‘counterfactual’) on forest converted to agriculture. While other studies might have different estimands of interest (e.g. Average Treatment Effects on the Treated (ATT), or even on the Untreated (ATU))^39^ our aim was to capture population-wide effects in order to broadly measure the influence of different tenure regimes across Brazil.

We faced the limitation that although exact-matching using CEM improved the balance in the data and the robustness of estimates, dropping non-matched observations limited the generalizability of effects exclusively to the matched subsample of data (i.e. meaning effect estimated would be average treatment effects on the matched sample (ATM)). Given our overarching aim to determine the generality of effects, we applied recently developed statistical methods that extend the generalizability of effects from a sample of data to a broader population ^40^. Thus, using these statistical techniques and ensuring data requirements were met ^39,40^, the matched data subsample resulting from the matching procedure was used to estimate effects that were generalizable to the broader, target population of all Brazilian land parcels (ATE).

We specifically used a weighting approach to thus extend effect estimates to the entire population of Brazilian land parcels – within each particular spatiotemporal tenure-regime comparison. For this, we first obtained a stratified representative sample of the entire population of land parcels (of each tenure-regime comparison, at each spatiotemporal scale considered) in order to facilitate subsequent computational processing times. We used the same covariates used for matching (i.e. elevation, slope, travel time to nearest city, human population, and area) to stratify the entire population of parcels and extract a representative sample. Then, using the matched-data subsets and the stratified representative sample of the entire population, we conducted a generalizability assessment of each of these tenure-regime comparisons at each scale considered using the *generalize* package in R ^40^. We calculated Tipton’s index of generalizability (T-index), a metric that describes levels of covariate similarity between two groups (i.e. here, the matched subset of data, and the entire population of land parcels) (Source Data file). T-index values range from 0-1, with values closest to 1 describing a population that is highly generalizable, and values under 0.5 are likely not generalizable because the two groups are too dissimilar.

After assessing generalizability, we generated weights in order for the matched subsample to more closely represent the entire population. Weights were calculated as the inverse odds of their probability of being matched, meaning that observations with a greater probability of being in the entire population had greater weights, and were obtained via lasso. Here, it is important to note that we trimmed the population to only include observations that did not exceed bounds of the matched covariates, in order to comply with the coverage assumption as a necessary condition to make further generalizations ^40^. To best characterize cases where matched data subsets were sufficiently different than the entire population of land parcels, we also calculated the absolute standardized mean difference (ASMD), of each covariate (Supplementary Table 8). Finally, in order to estimate ATE, weights were incorporated into subsequent regression models using the uncoarsened matched-data subset (see section 3.5).

Note, in a few cases (4 undesignated/untitled models in Caatinga (Source Data file)), T-index calculations failed due to a low sample size of the entire population of land parcels, preventing any statement on the generalizability of these cases to their entire populations. While weights were still generated and included in the final statistical models (see 3.5), effect estimates may not be generalizable to the entire population of land parcels in these cases, but only apply to the (weighted) matched-data subsample.

**3.5. Regression analyses**

For each scale and tenure-regime comparison, we estimated effects by fitting generalized linear models (GLMs) with a binomial error distribution and a logit link to the respective matched dataset. We used the uncoarsened variables as model covariates, previously generated weights, and additionally included federal state as a fixed-effect to control for state-level differences in governance regimes and effectiveness. To control for possibly remaining spatial autocorrelation in model residuals, we cluster our standard errors by municipality.

(1) $logit\left( p \right)= \beta_{0}+ \beta_{1}tf+ \beta_{2}l+\beta_{3}s+ \beta_{4}tt+ \beta_{5}pd+ \beta_{6}r+ \beta_{7}w+ \beta_{7}st$

where *p* is the per-pixel probability of forest conversion, *tf* is the tenure regime, *l* is the average elevation in meters, *s* is the average slope in degrees, *tt* is the average travel time to nearest city in minutes, *pd* is the average population density, *r* is the area of the parcel in ha, *w* is the generated weights, and *st* the federal state. Note that binomial models of percentage forest loss automatically capture differences in initial forest area, by evaluating the total forest areas (counts of pixels) that were converted to agriculture vs. those that remained. We calculated average marginal effects (AME) using the ‘*margins’* package in R ^41^, transforming coefficient estimates to average per-forest-pixel probability of conversion to agriculture with respect to the tenure form in question ^42^ (Source Data file).

Note that in rare cases, insufficient observations distributed across federal states prevented the estimation of coefficients for all parameters in those models. We addressed this by consecutively merging geographically-adjacent states until parameters could be estimated, keeping the merging protocol as consistent as possible across models (see Supplementary Table 3). This merging of states allowed for the correct estimation of parameters in 3 models. However, the model still failed to converge in the remaining 3 models, likely due to the insufficient number of observations distributed across federal states causing a pattern in the data commonly known as complete separation ^43,44^. While this kind of convergence issue in logistic regression is well known ^43,44^, achieving model convergence for these cases would likely require using a different modeling approach, and could involve excluding federal state as a variable in the model ^44^. We maintained the modeling approach that was most appropriate for the vast majority of data in this analysis, and report models with convergence issues (Source Data file, Supplementary Table 3)^45^.

Lastly, we calculated Rosenbaum bounds as a sensitivity analysis to assess whether our model estimates are robust to the possible presence of omitted-variable bias. Rosenbaum bounds quantify the sensitivity of our regressions results to different magnitudes of hypothetical bias that might be caused by missing important confounders in the matching procedure ^46^. Here, the magnitudes of bias (Γ) are expressed as the change in the odds of being selected into treatment or control caused by the addition of a hypothetical unobserved confounder. We calculated lower and upper bounds for both Hodges-Lehmann point estimates and *p*-values (see supplementary files) using the ‘*rbounds’* package in R. Our calculations showed that both Hodges-Lehman estimates and *p*-values were not highly sensitive to possible small omitted-variable bias (Γ = 1.1), and were still reasonably robust to possible large omitted-variable bias (Γ = 1.5). Across tenure-regime comparisons, spatial scales, and temporal scales, average sensitivities of estimated effects ranged from, respectively, 11.18%, 10.12% and 10.78% relative error at Γ = 1.1, to 48.72%, 44.48% and 46.92% at Γ = 1.5 (Supplementary Table 6; relative error calculated as percentage of the magnitude of the respective median effect size at Γ=1). Average sensitivities of significance of effects (p ≤ 0.05) ranged from, respectively, 2.7%, 4.2% and 3.2% of models with a sensitive effect significance at Γ = 1.1, to 17.3%, 15.6% and 18.11% at Γ = 1.5 (Supplementary Table 6). We did not find any systematic patterns in sensitivity to possible omitted-variable bias across tenure-regime comparisons, regions, or time periods, except that results based on lower sample sizes (mainly comparisons involving quilombola tenure and those in the Caatinga biome) were on average slightly more sensitive. Our analysis implies that the magnitude of estimated differences in outcomes between treatment and control units, and their significance, is only slightly sensitive to the possibility of a missing confounder, if present. We note that this sensitivity test cannot indicate whether or not an unobserved-confounder bias is actually present.

**3.6. Cross-scale synthesis of effects**

To assess which statements on deforestation effects of tenure-regime differences might be transferable across diverse socio-environmental contexts (e.g., different environmental settings, time periods, or administrative levels), we synthesized the scale-specific effects in two ways. First, for each comparison (e.g., private vs. undesignated/untitled), we assessed the consistency of the direction of the causal effect by calculating percentages of scale-specific models with, respectively, significant deforestation-increasing (positive), significant deforestation-decreasing (negative), and no significant effects (Supplementary Table 3). These analyses address the applied question of how reliably a particular tenure-regime change might decrease long-term deforestation rates under different (e.g., unknown, or unforeseeable) socio-environmental contexts. Second, we assessed the consistency of the relative ranking of alternative tenure regimes by the magnitudes of their effects vis-a-vis a given counterfactual, by calculating percentages of scales at which each tenure regime showed higher/lower effects than all others (Supplementary Table 3). These analyses address the applied question of which of alternative tenure-regime changes might most/least reliably cause *large* reductions in deforestation. Note that, although these relative rankings indirectly compare alternative tenure regimes to differently-matched counterfactuals, as a part of the analysis that extends the generalizability of effect estimates, both undesignated/untitled and private counterfactuals were weighted to represent the covariate distribution in the entire population of parcels at each respective scale evaluated. This weighting effectively provided a standardized counterfactual for all estimations across tenure-regime comparisons at different scales.

We had initially considered using formal meta-analyses as a third way of synthesizing the scale-specific effects, which would have indicated the direction and magnitude of ‘average’ effects. However, testing indicated high heterogeneity, which, in combination with our small sample sizes (i.e., numbers of scale-specific models) precluded us from deriving reliable estimates using meta-analyses ^47^.

We assessed the robustness of the results of our cross-scale synthesis against possible bias in the relative reliability of the tenure-comparison- and scale-specific causal tests. To this end, we additionally calculated balance-weighted percentages that effectively downweigh any cases where covariate overlap post-matching remained low, and based all our main conclusions on qualitatively consistent balance-weighted/unweighted results. Specifically, we calculated balance-weighted percentages of cases with significant-negative, significant-positive, and nonsignificant effects by weighting each tenure-comparison- and scale-specific result contributing to a given percentage value by the inverse of the remaining imbalance (*L_1_*) in the respective dataset (Supplementary Tables 3-5). Similarly, we calculated weighted percentages of scales at which each tenure category had higher/lower-ranked effects than all others by weighting the entire set of tenure-regime comparisons contributing to the ranking at a given scale by the inverse imbalance (*L_1_*) of the least-balanced dataset at that scale (Supplementary Table 3). In addition to this balance-weighting, we also assessed the robustness against violations of the assumption of constant treatment of parcels with strict-protection and sustainable-use regimes (see section 2.2), by using results based on time-filtered datasets to calculate alternative versions of percentages with significant-negative, significant-positive, and nonsignificant effects (Supplementary Table 4; see Supplementary Fig. 3-4 and Source Data file for the full time-filtered results; see section 2.2 for explanation of time-filtering).

We also assessed whether differences in how often tenure regimes were ranked as most/least effective in reducing deforestation might be biased by systematic differences in the different regimes’ exposures to deforestation pressures. Such bias would in principle be possible, as these assessments of relative effectiveness are based on comparisons among the regimes’ effect sizes at each scale, which were all estimated with unique combinations of matched parcels. In particular, we expected the indirect comparison of strict-protection vs. sustainable-use regimes (vis-a-vis an undesignated/untitled counterfactual) to be potentially affected by differences in geographical siting of the different types of conservation areas relative to deforestation pressures, which has been previously reported for Amazonia ^48^. We thus assessed whether their differing percentages of most/least effective cases reflected systematic differences in their matched parcels’ average covariate values at the specific scales where they were most/least effective. While we did find some cases where the two tenure regimes differed with respect to specific covariates, these cases did not indicate any systematic bias. For example, strict-protection regimes were often ranked as less effective in reducing deforestation than sustainable-use areas in the Amazonia and Mata Atlântica biomes, despite occurring in, respectively, more remote, and higher-elevation areas on average (cf. ^19^.

**3.7 Assessment of potential bias due to differences in initial forest cover**

We note that the estimated effects of tenure-regime differences could have been affected by differences in initial forest cover between our matched parcels that resulted from forest-to-agriculture conversions prior to the respective treatment periods. In particular, forest conversion rates on private lands might change with decreasing forest cover, as the Forest Code prohibits additional deforestation once forest cover decreases to a certain threshold (e.g. 80% in the Amazonia biome). Similarly, parcels in old deforestation frontiers might have already been past their deforestation peaks before our study periods began, whereas those in newly emerging frontiers might not yet experience the magnitude of deforestation that is this yet to come.

To assess possible bias in our conclusions due to systematic differences in initial forest cover, we modelled the initially forest-covered percentages of the matched parcels’ areas at each spatiotemporal scale as a function of their treatment (i.e., tenure-regime identity). To this end, we fitted GLMs with a binomial error distribution and a logit link to the respective matched datasets to estimate the per-pixel likelihood of being initially forest-covered. Beyond a dummy variable distinguishing treatment and control, we included all covariates from our main regression analyses to compare the same parcels that were also originally matched (see 3.3). We detected no systematic unidirectional differences between treatment and control across scales, indicating that our main conclusions are not biased by such differences (see Supplementary Fig. 6). However, we found differences in either direction in individual cases and thus cannot rule out that these might partly explain differential forest trajectories for some tenure regimes and spatiotemporal scales. We addressed this caveat by basing our main conclusions on results that showed consistency across spatiotemporal scales and by ruling out this bias when drawing insights from scale-specific results (e.g., the changing relative effectiveness of tenure regimes in curbing Amazonian deforestation).

We chose this indirect approach over directly matching parcels on initial forest cover. This was motivated, firstly, by our aim to evaluate all tenure regimes via a consistent modelling protocol. Here, retaining sufficient degrees of freedom for each tenure regime and spatiotemporal scale required us to constrain the total number of matching covariates, as that number affects both the matched dataset sizes and the number of modelling covariates included in the binomial GLMs. Secondly, our specific aim was not to assess total forest losses of different tenure regimes over their entire lifetimes (which would necessitate accounting for any prior deforestation already internalized in parcels’ initial forest cover), but to assess whether tenure regimes consistently differed in their ability to retain remaining forest cover over different time periods (defined by their unique historical deforestation trends, policies, etc.). Here, differences in the magnitude of additional percentage losses among the matched parcels are already internalized in the way percentages are modelled by binomial GLMs. Finally, parcel-level differences in initial forest cover do not necessarily reflect prior forest-to-agriculture conversions, but may also reflect natural spatiotemporal heterogeneity in land cover (e.g., due to mosaics of forest and non-forest vegetation, landslides, etc.) as well as earlier agricultural expansion over non-forest vegetation, particularly outside the Amazonia biome.

Supplementary Figures


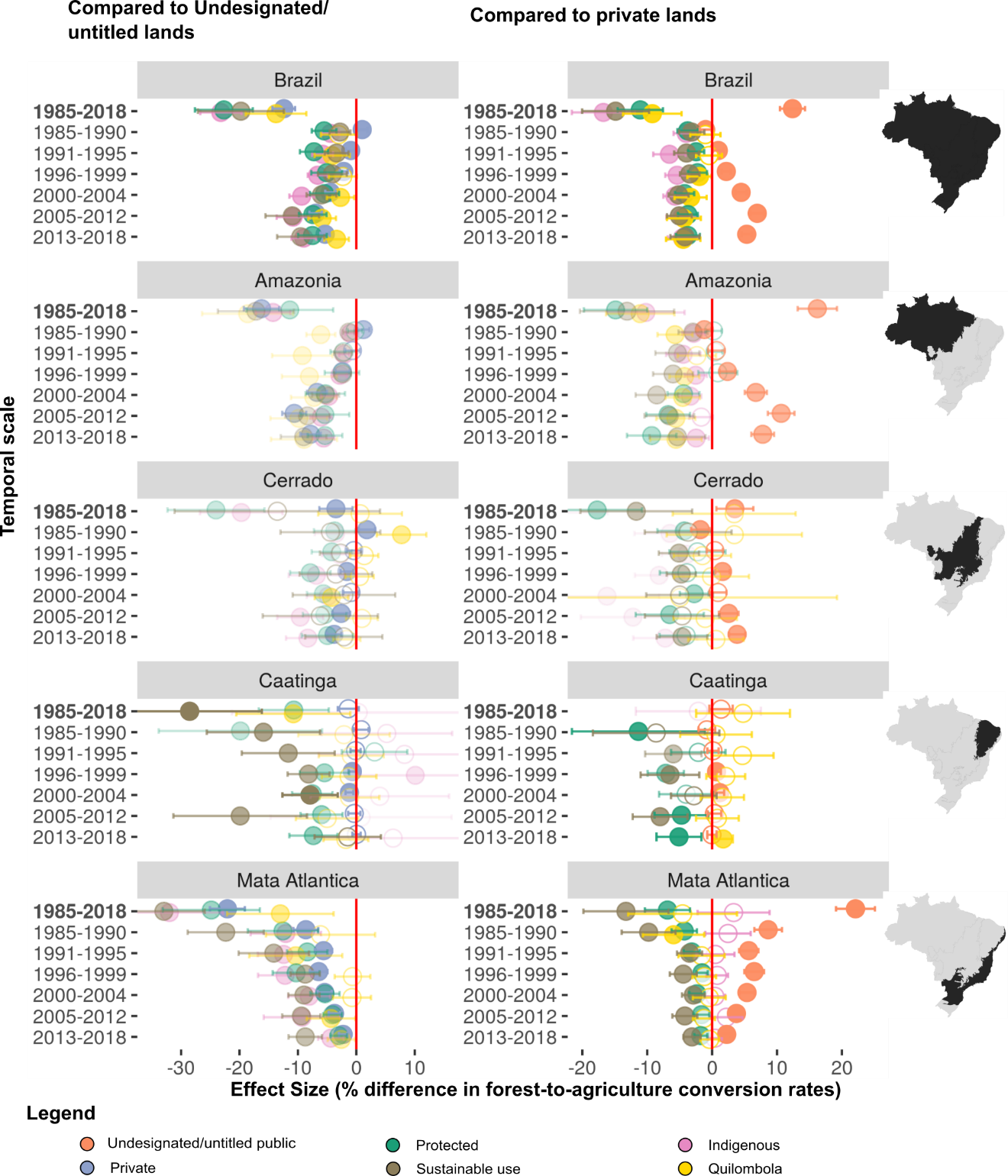


Supplementary Fig. 1. Effects of alternative land-tenure regimes on forest-to-agriculture conversion rates in Brazil, disaggregated to different spatiotemporal scales. Circles indicate average effects sizes estimated using regression analysis at the respective scale vis-a-vis two alternative counterfactuals: a) undesignated/untitled public lands, and b) private lands. Effects to the left of the zero line indicate a decrease in average parcel-level deforestation rate (to the right: increase). Filled circles indicate statistically significant effects (p ≤ 0.05; non-filled: not significant), upper/lower confidence intervals are plotted to the left/right of each circle centroid. Higher transparency of filled circles indicates high levels of imbalance in the matched dataset (multivariate imbalance measure *L_1_*).


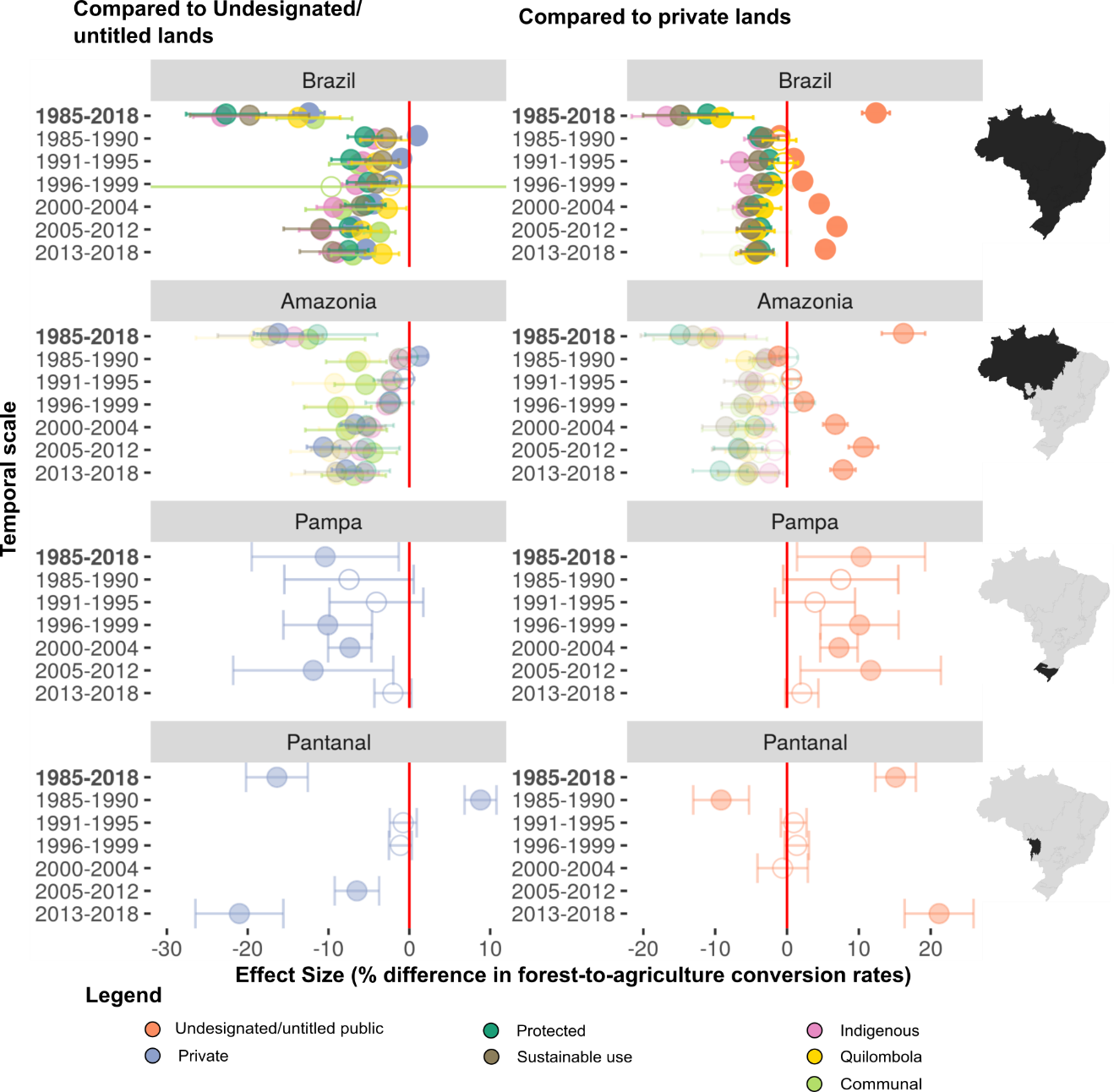


Supplementary Fig. 2. Effects of alternative tenure regimes on forest-to-agriculture conversion rates at different spatiotemporal scales, complementing Supplementary Fig. 1 by showing additional results for communal tenure regimes for Brazil and the Amazonia biome, and for private and undesignated/untitled regimes for Pampa and Pantanal. Circles indicate average effects sizes estimated using regression analysis at different spatial-temporal scales, where each tenure regime was compared vis-a-vis two alternative counterfactuals: a) undesignated/untitled public lands, and b) private lands. Effects to the left of the zero line indicate a decrease in average parcel-level deforestation rate (to the right: increase). Filled circles indicate statistically significant effects (p ≤ 0.05; non-filled: not significant); upper/lower confidence intervals are plotted to the left/right of each circle centroid. Higher transparency of filled circles indicate high levels of imbalance in the matched dataset (multivariate imbalance measure *L_1_*). Note that tests for communal tenure had to be based on substantially fewer parcels than those for other tenure regimes, with sufficient parcels post-matching for reliable parameter estimation only available at the Brazil-wide and Amazonia-wide scales. Similarly, the only reliable comparison possible in the Pampa and Pantanal biomes was undesignated/untitled vs. private, due to a lack of data for other regimes (and/or lack of certain tenure regimes) in these biomes.


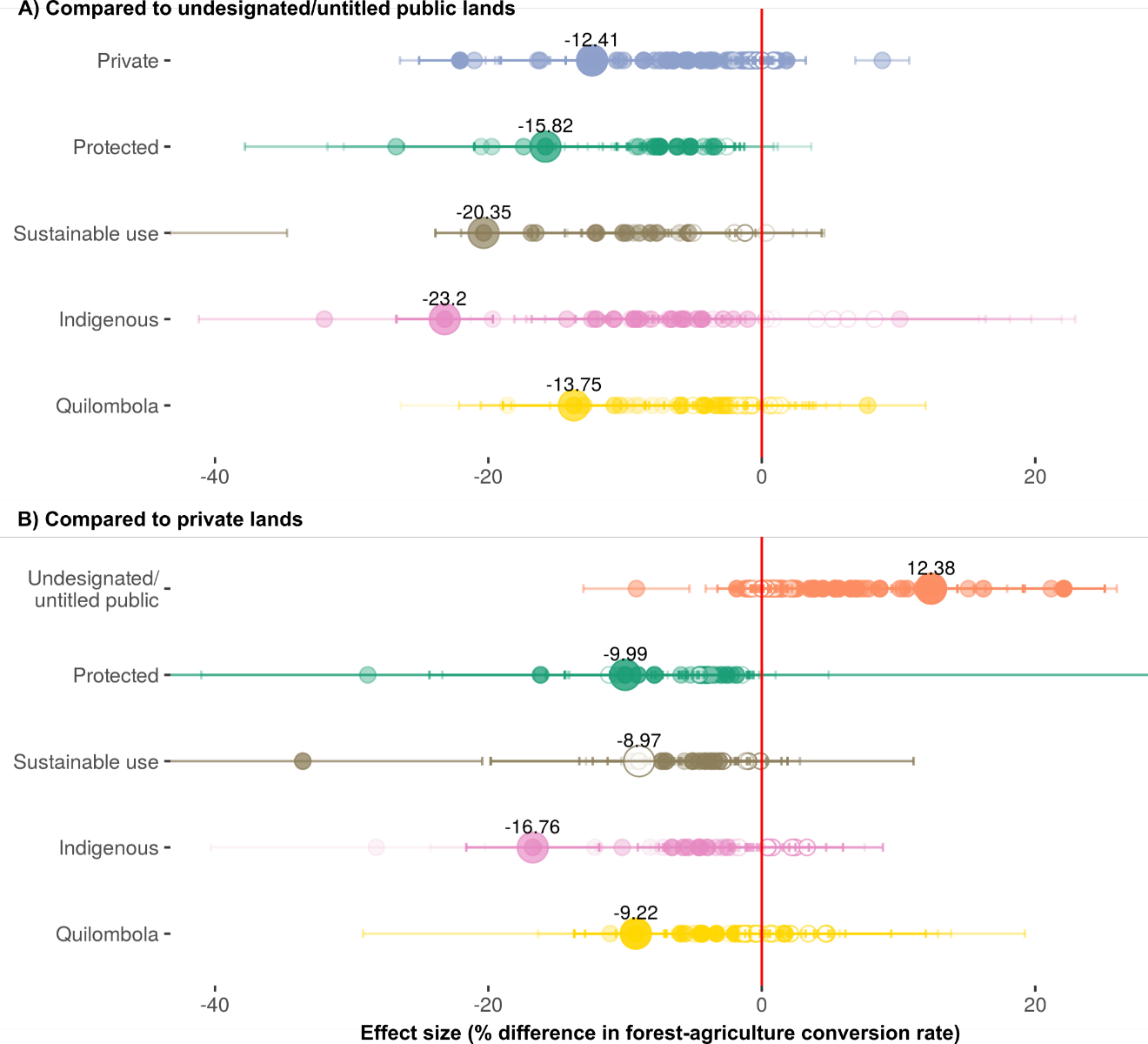


Supplementary Fig. 3. Robustness test of effects of alternative tenure regimes on forest-to-agriculture conversion rates in Brazil using filtered time-series data for protected and sustainable-use areas (i.e., only areas established before/during beginning of each temporal scale considered; see section 2.2). Circles indicate average effect sizes estimated using regression analysis at different spatial-temporal scales vis-a-vis two alternative counterfactuals: A) undesignated/untitled public lands, and B) private lands. (see Supplementary Fig. 4 for detailed presentation). Labelled effect sizes (larger circles) report effects across Brazil over the time period 1985-2018, Effects to the left of the zero line indicate a decrease in average parcel-level deforestation rate (to the right: increase). Filled circles indicate statistically significant effects (p ≤ 0.05; non-filled: not significant); upper/lower confidence intervals are plotted to the left/right of each circle centroid. Higher transparency of filled circles indicate high levels of imbalance in the matched dataset (multivariate imbalance measure *L_1_*).


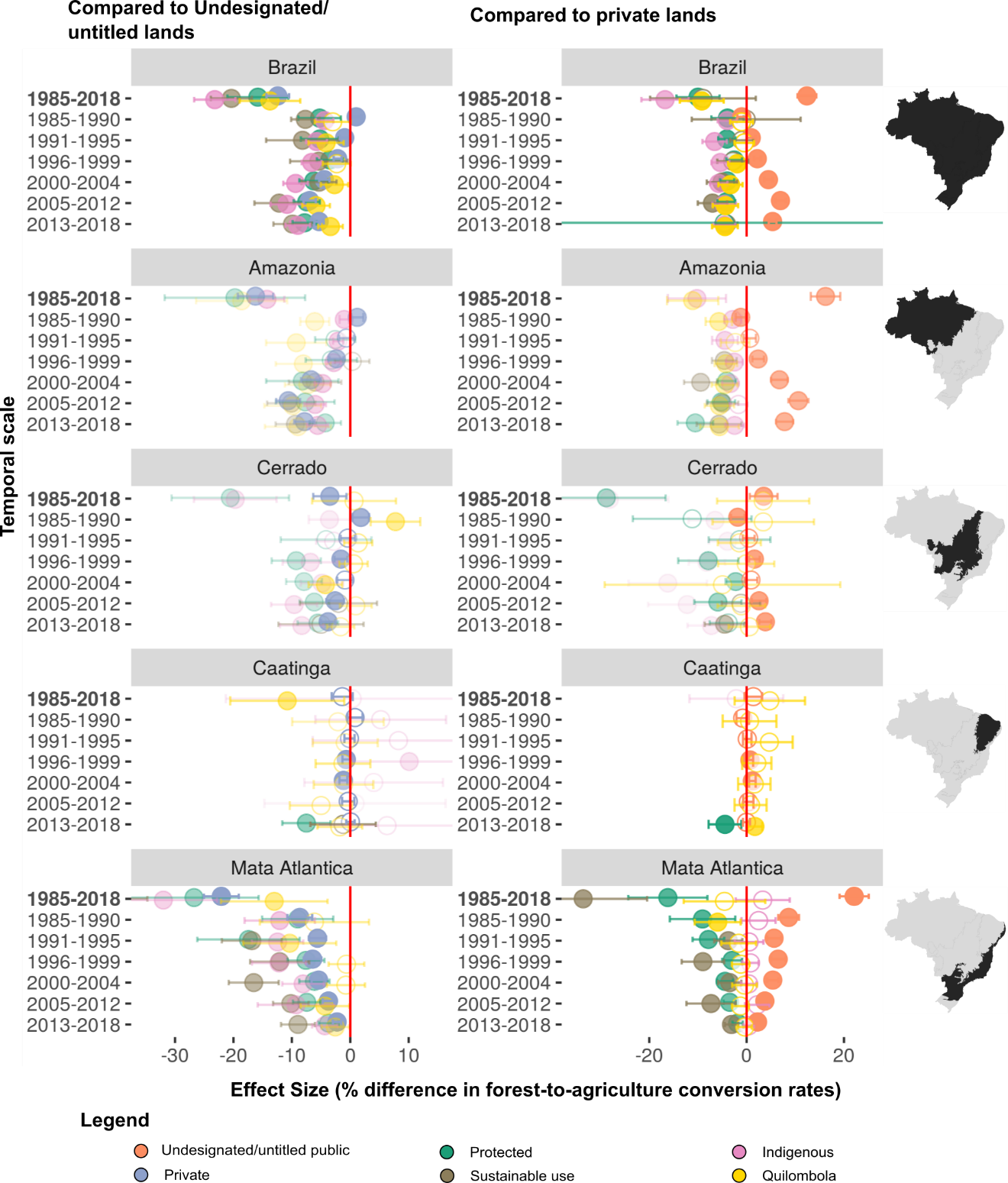


Supplementary Fig. 4. Spatiotemporal disaggregation of robustness test of effects of alternative tenure regimes on forest-to-agriculture conversion rates in Brazil using filtered time-series data for protected and sustainable-use areas (i.e., only areas established before/during beginning of each temporal scale considered; see section 2.2). Circles indicate average effect sizes estimated using regression analysis at different spatial-temporal scales vis-a-vis two alternative counterfactuals: a) undesignated/untitled public lands, and b) private lands. Effects to the left of the zero line indicate a decrease in average parcel-level deforestation rate (to the right: increase). Filled circles indicate statistically significant effects (p ≤ 0.05; non-filled: not significant), upper/lower confidence intervals are plotted to the left/right of each circle centroid. Higher transparency of filled circles indicate high levels of imbalance in the matched dataset (multivariate imbalance measure *L_1_*).


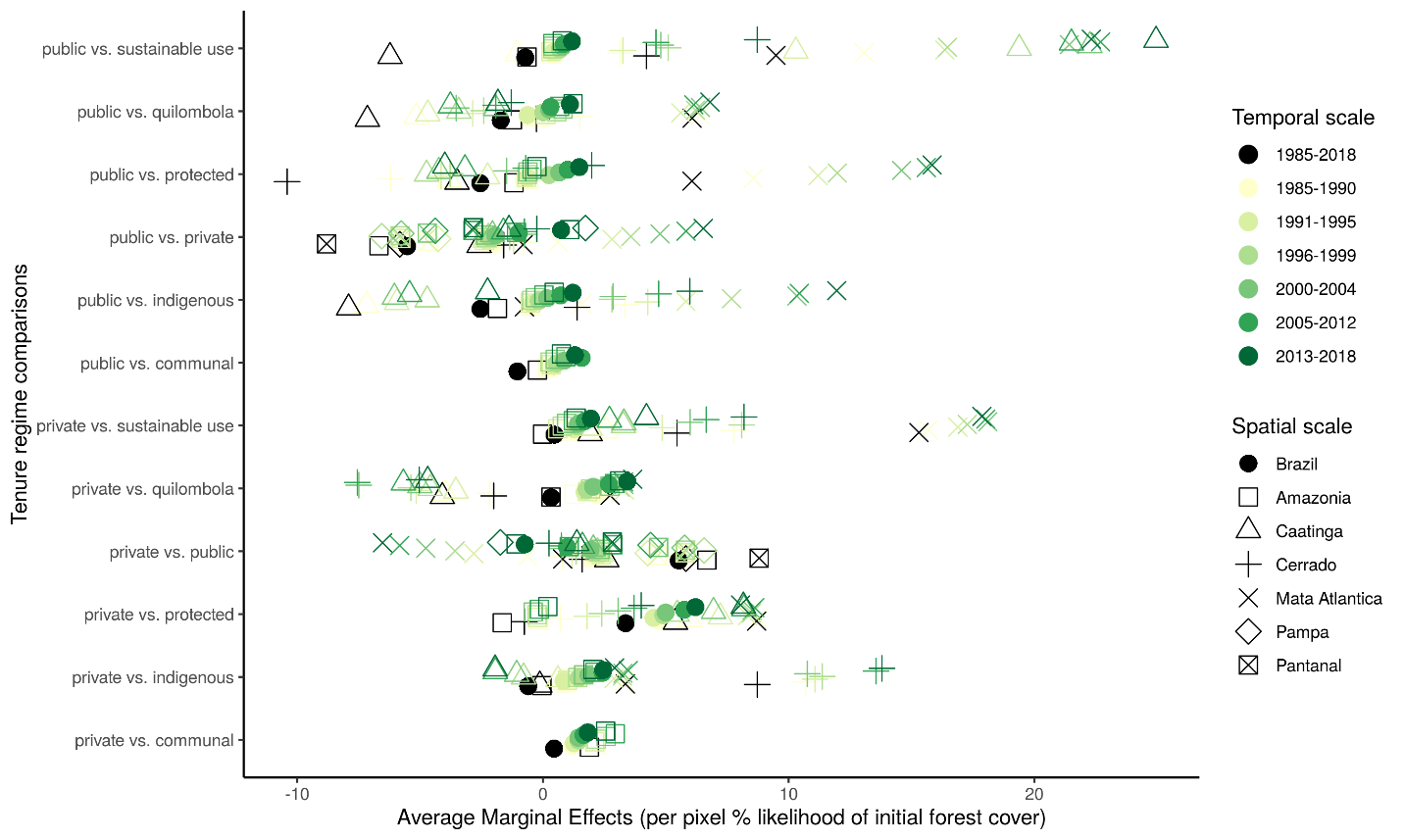


Supplementary Fig. 5. Differences in initial forest cover between matched treatment and control units for different tenure-regime comparisons at different spatial and temporal scales. Average marginal effects indicate the per-pixel likelihood being forest-covered at the beginning of each time period considered. At the parcel level, these can be interpreted as average deviation in initial percentage forest cover of the parcels treated with a given tenure regime relative to their matched counterfactual parcels. Temporal scales and spatial scales are indicated by color and shape, respectively, with broader scales (Brazil, 1985-2018) indicated in black. Symbols clustering closely around 0 and/or deviating from 0 in either direction indicate that the cross-scale synthesis results are unlikely biased by systematic differences in initial forest cover.

Supplementary Tables

Supplementary Table 1. Non-exhaustive overview of hypotheses linking land tenure to deforestation, along with their predictions on the direction and relative strength of effects of different land-tenure regimes on deforestation rates. The top group of hypotheses (‘Bundles of Rights’) are classified by the rights dimension that they mainly address, either directly or through a series of mechanisms, and the bottom group (‘Cross-cutting themes’) relates to other tenure-related aspects. Arrows indicate predicted increases/decreases of deforestation of a shift from either undesignated/untitled (left) or private lands (right) to each alternative tenure regime. Arrows follow a six-point scale, with the dark green downward-pointing arrow indicating the strongest predicted decreases in deforestation and the dark red upward-pointing arrows indicating the strongest increases. Note that these are *ceteris paribus* predictions, assuming that the specified mechanisms would affect deforestation rates in isolation, rather than in an interplay of multiple mechanisms. Also note that these predictions reflect the specific bundles of rights associated with land-tenure regimes in Brazil (see Table S2). Because not all hypotheses are relevant to all comparisons, some cells are left blank.

| **Thematic dimension** | **Hypothesized mechanism** | **References** | **Predicted effect of tenure regime on deforestation,  relative to undesignated/untitled public lands** | | | | | | **Predicted effect of tenure regime on deforestation,  relative to private lands** | | | | | |
| --- | --- | --- | --- | --- | --- | --- | --- | --- | --- | --- | --- | --- | --- | --- |
|  |  |  | Private | Protected area | Sustainable use | Indigenous | Quilombola | Communal | Undesignated/untitled c | Protected | Sustainable use | Indigenous | Quilombola | Communal |
| **Bundle of Rights** | |  |  |  |  |  |  |  |  |  |  |  |  |  |
|  | |  |  |  |  |  |  |  |  |  |  |  |  |  |
| Exclusion | Open-access, common-pool resources are by definition non-excludable. Low exclusion rights will increase deforestation through unsustainable use by multiple competing resource users ^49–51^. Undesignated/untitled public lands lack both clear supervision by any designated agency and effective exclusion rights, making them often de-facto open access environments. Traditionally, community-based tenure regimes have been viewed as facing similar challenges in excluding outside users due to different impediments to collective action ^52,53^. | Gordon, 1954; Hardin 1968; Browder & Godfrey, 1997; Grafton 2000; Sandler 2015 | **↘** | **↘** | **↘** | **↘** | **↘** | **↘** | **↗** | **→** | **↗** | **↗** | **↗** | **↗** |
| Alienation | Alienation rights allow tenants to use land as collateral in business transactions and to access credit, thus providing them larger financial means to engage in forest-displacing agricultural activities. By contrast, land without alienation rights (e.g. untitled public lands, indigenous lands, and quilombola lands) do not provide these options, thus inhibiting investments in deforestation-promoting land uses ^54,55^. | de Soto 2000; Place and Otsuka, 2002 | **↗** | **↘** | **↘** | **↘** | **↘** | **↗** | **↘** | **↘** | **↘** | **↘** | **↘** | **↘** |
| Alienation | Under sufficiently functioning land markets, rights to rent out or sell land will eventually result in lands being transferred to those entities who can put them to the financially most productive use, which will often be a non-forest use ^56^. | Deininger et al., 2003 | **↗** | **↘** | **↘** | **↘** | **↘** | **↗** | **↘** | **↘** | **↘** | **↘** | **↘** | **↘** |
| Alienation and withdrawal rights | Only land that can be legally be sold or otherwise alienated by the current tenant is potentially available to people searching for land for farming (mainly private, and to a lesser extent communal and undesignated/untitled lands). Because the expected higher agricultural profits enabled by commercial withdrawal rights tend to be factored into land prices for private lands on formal land markets, these are often unaffordable to poor smallholders or land-less settlers searching for land. These will thus instead be forced to settle on undesignated public lands at the 'frontier' ^57^. | Binswanger, 1991 | **↘** | **↘** | **↘** | **↘** | **↘** | **↘** | **↗** | **↘** | **↘** | **↘** | **↘** | **↗** |
| Withdrawal and market integration | Tenure forms that grant commercial withdrawal rights are economically more capable of engaging in high-input land-uses, facilitating deforestation at comparatively larger scales. This effect is stronger if tenants are more capable of commercializing their resources through greater market integration ^58^. | Anderson, 2018 | **↗** | **↘** | **↗** | **↘** | **↗** | **→** | **↘** | **↘** | **↘** | **↘** | **↘** | **↘** |
| Withdrawal and perceived tenure security (e.g., through private titles) | Tenure forms with commercial withdrawal rights and high perceptions of tenure security provide greater incentives to engage in forest-displacing land-use activities (e.g., cropping or cattle ranching). For example, private tenure, with both commercial withdrawal rights and often higher tenure security, should thus lead to higher deforestation rates compares to undesignated/untitled lands, where commercial withdrawal is unregulated or encouraged, but there is little assurance of future benefits from current investments in land-use ^59^. | Liscow, 2013 | **↗** | **↘** | **↘** | **↘** | **↘** | **↘** | **↘** | **↘** | **↘** | **↘** | **↘** | **↘** |
| Withdrawal (non-commercial) | Deforestation through subsistence use is most likely to occur in contexts where land users are dependent on unsustainably exploiting their forest resources for their short-term survival (e.g., during climate-induced resource shortages and in absence of alternative livelihood options)^60^. Where this is the case, tenure regimes with highly restricted or no withdrawal rights for subsistence (mostly fully protected areas) will have lower deforestation rates than all those with withdrawal rights for subsistence. Among those tenure regimes, those that *only* grant withdrawal rights for subsistence (e.g., indigenous), will have higher rates of deforestation compared to those tenure regimes that grant tenants restricted commercial withdrawal rights (e.g., quilombola, communal, sustainable-use areas) and those that do not explicitly prohibit commercial exploitation (e.g., rural settlements on public lands). Those tenure regimes that enable full integration into markets (private properties) will least strongly affect forest resources via subsistence withdrawal, as the latter regimes provide better options for alternative (non-subsistence-withdrawal) ways of sustaining livelihoods. | Perrings, 1989 | **↘** | **↘** | **↘** | **↗** | **↗** | **↗** | **↗** | **↘** | **→** | **↗** | **↗** | **↗** |
| Withdrawal (commercial and non-commercial) | Tenure regimes where resource withdrawal is either not restricted or incentivized will see higher deforestation rates ^61–63^. For example, undesignated/untitled public lands will often have higher deforestation, as governments rarely place restrictions of deforesting them, or even incentivize it by granting land claims based on prior clearance of forest, or by allowing settlement conditionally on putting the land to productive (i.e., agricultural) use. | Angelsen, 1999; Fearnside, 2005; Redo, 2011 | **↘** | **↘** | **↘** | **↘** | **↘** | **↘** | **↗** | **↘** | **↘** | **↘** | **→** | **↘** |
| Withdrawal (commercial and non-commercial) | Tenure regimes that grant but regulate rights to withdraw forest resources incentivize tenants to manage these resources for long-term sustainability, leading to lower deforestation rates compared to regimes with no or more unregulated withdrawal rights ^64–68^. | Nepstad et al., 2006; Bray et al., 2008; Ellis and Porter-Bolland, 2008; Duchelle, 2012; Porter-Bolland et al., 2012 | **↘** | **→** | **↘** | **↘** | **↘** | **↘** | **↗** | **↗** | **↘** | **↘** | **↘** | **↘** |
| Exclusion & due process (or other mechanisms increasing tenure security) | Tenure forms with stronger exclusion rights, together with due-process rights or other mechanisms that provide tenure security, create the highest incentives for investments in the resource, by providing assurance that the later benefits from resource withdrawal or other exploitation can be enjoyed exclusively ^69–71^.  Thus, tenure forms with greater assured exclusivity of resource rights are expected to lead to the allocation of land to the use form of greatest long-term economic utility to the tenant. This will commonly be agricultural uses in private farms and public rural settlements, and forest uses in protected areas, sustainable use areas, and indigenous reserves, with more ambiguous outcomes expected for other community-based tenure regimes. | Birdyshaw and Ellis, 2007; Deacon_et al., 1994; Deininger et al., 2003 | **↗** | **↘** | **↘** | **↘** | **↘** | **↗↘** | **↘** | **↘** | **↘** | **↘** | **↘** | **↘** |
| Types of tenants and main rights holders | Traditional communities collectively holding land (e.g. indigenous, quilombola, and other communities with traditionally-rooted land-tenure regimes) typically create societal rules to effectively manage common forest resources and govern their use. Community members tend to follow these rules to avoid social exclusion, leading to reduced degradation of communally regulated forest resources, relative to state-managed resources ^72–74^.  Undesignated/untitled public lands are expected to have higher rates of deforestation than indigenous, quilombola, and communal lands. | Mendelsohn and Balick, 1995; Gibson et al., 2000; Baland and  Platteau 2000 |  |  |  | **↘** | **↘** | **↘** |  |  |  |  |  |  |
| Exclusion | In contexts where the holder of monitoring, enforcement, or other duties has limited capacity to meet these duties, excludability is impaired. In low-governance regions, where public institutions have limited capacities, tenure regimes where the state is the main duty holder should thus have higher deforestation rates than tenure regimes where local tenants are responsible for these duties ^61,52,62,75^. Among the latter regimes, the ability to fulfill these duties and thus effectively exclude intruders should increase with the number of people available for these tasks (e.g., higher for quilombola communities than for individual private tenants). | Angelsen, 1999; Grafton, 2000; Fearnside, 2005, Nolte et al. 2013 | **↘** | **→** | **→** | **→** | **↘** | **→** | **↗** | **↗** | **↗** | **↗** | **↘** | **↗** |
| **Cross-cutting themes** | |  |  |  |  |  |  |  |  |  |  |  |  |  |
| Number of resource users and/or decision-makers | Decision making regarding the use and conversion of forests have higher transaction costs in community-based tenure forms because it takes more time and resources to reach decisions with larger numbers of people ^76,77^. Individuals or small groups, in turn, have lower transaction costs involved in this decision-making process, meaning that they are more agile in responding to economic pressures or incentives to allocate the land to its most profitable use (which in many contexts implies converting forest to cropland or cattle ranching).  Thus, tenure regimes with higher numbers of resource decision-makers are expected to decrease deforestation compared to those with lower numbers of decision-makers | Naidu 2009; Ostrom, 2009 | **↗** | **→** | **→** | **↘** | **↘** | **↘** | **↘** | **↘** | **↘** | **↘** | **↘** | **↘** |
| Number of resource users and/or decision-makers | Tenure regimes where ownership is shared among larger numbers of people are better equipped to monitor and protect their land, decreasing the likelihood of deforestation as compared to properties with fewer people ^76,78^.  Thus, tenure regimes with higher numbers of owners, resources users, or decision-makers are expected to decrease deforestation compared to tenure forms with fewer numbers. | Sakurai et al., 2004; Ostrom 2009 | **↗** | **→** | **→** | **↘** | **↘** | **↘** | **↘** | **↘** | **↘** | **↘** | **↘** | **↘** |
| Number of resource users and/or decision-makers | Tenure regimes with higher numbers of individual users are expected to be more likely to unsustainably exploit forest resources for individual short-term gain and thereby cause the collapse of the resource system than tenure forms with few or one user(s)^49,51,79^. | Gordon, 1954; Browder et al., 1997; Klingler and Mack, 2020 | **↘** | **→** | **→** | **↗** | **↗** | **↗** | **↗** | **↗** | **↗** | **↗** | **↗** | **↗** |
| Tenure security | Low levels of tenure security are commonly viewed as inhibiting tenants' engagement with their land resources (e.g., investment) due to elevated risk that all or some tenure rights may be cut short before they see the benefits of their investment ^80^. Higher levels of tenure security are thus classically expected to incentivize users to more readily ‘invest’ in increasing the profitability of the land resource. In most tropical forestland contexts, this hypothesis would predict these to be investments into allocating the land to a more profitable use (e.g., through a conversion of forest to cropland or cattle ranching), but these may also be investments into, e.g., restoring a degraded land resource. By contrast, lower levels of tenure security may also be expected to increase deforestation-causing activities if land clearing is used to solidify claims on the land ^61,62,81,82^. While private land tenure is classically viewed as providing the highest tenure security and thus assurance levels, this view is not universal ^83^.  Assuming that classical views on tenure-form–tenure-security relationships broadly hold and that landholders are mainly economically/personal-survival motivated, this set of hypotheses would predicts a skewed u-shaped relationship between tenure security and deforestation rates, where deforestation is medium-high at very low tenure security levels (e.g., informal settlements on public lands), lowest at intermediate levels of tenure security (i.e. indigenous, quilombola, and communal lands), and highest under highest assurance levels (e.g. private tenure). | Holden and Yohannes, 2002; Angelsen, 1999; Fearnside, 2005; Deininger and Jin, 2006; Fenske, 2011; Robinson et al., 2004 | **↗**  **↘** |  |  | **↘** | **↘** | **↘** | **↗**  **↘** |  |  | **↘** | **↘** | **↗**  **↘** |
| Governance (monitoring and enforcement) | Tenure regimes where the state (i.e., citizenry) is the main or exclusive rights and duty holder, such as protected areas or other lands administered by public institutions, are expected to have lower deforestation rates than other tenure regimes because the state is more likely to benefit from economies of scale for monitoring, enforcing, processing of information, and other management-related activities that prevent deforestation ^52^. | Grafton, 2000 | **↗** | **→** | **→** | **→** | **↗** | **→** | **↘** | **↘** | **↘** | **↘** | **→** | **↘** |
| Governance (monitoring and enforcement) | Tenure forms where a single entity is the main rights holder (i.e., private tenure) provide better opportunities for state or federal agencies to enforce environmental legislation than tenure forms where the main rights holder is a community, unknown, or abstract (e.g., citizenry) because this increases accountability in adhering to targeted environmental legislation meant to decrease deforestation. Thus, tenure forms where single entities are the main rights holders are expected to decrease deforestation in comparison to those with multiple entities as rights holders ^84,85^. | Hargrave and Kis-Katos, 2013; Arima et al. 2014 | **↘** |  |  |  |  |  | **↗** | **↗** | **↗** | **↗** | **↗** | **↗** |
| Governance (monitoring and enforcement) | In countries with a history by short-lived government institutions or volatile political directions, government programs proposing investments in the long-term sustainability of forest resources will lack credibility. Therefore, publicly owned forests will not be used sustainably, even if these are under partial private or community-based management ^86^. | Deacon, 1994 | **↘** | **→** | **→** | **→** | **↘** | **→** | **↗** | **↗** | **↗** | **↗** | **→** |  |
| Governance | Public institutions in countries with poorly developed governance systems and/or high levels of external debt are more likely to sell or lease rights to exploit national resources (e.g., forestlands) at abnormally low prices. This increases the likelihood of inefficient, resource-intensive land-use forms (e.g. agricultural expansion rather than intensification). In such contexts, resource users are also more likely to overexploit resources (whether sold or leased) beyond the legal limit allowed because the perceived likelihood of enforcement is low ^74^. Thus, under precarious governance contexts, all publicly owned forestland is expected to be more likely to experience deforestation. | Baland and Platteau, 2000 | **↘** | **→** | **→** | **→** | **↘** | **→** | **↗** | **↗** | **↗** | **↗** | **→** |  |

Supplementary Table 2. Tenure regimes in Brazil and associated bundles of rights. We re-categorized 14 land-tenure categories distinguished in Brazil (first column) into seven tenure regimes (second column). For each regime, we defined the typical number of tenants involved in land decision-making (third column), as well as the main types of rights holders (who hold this particular bundle of rights) and main duty holders (who are responsible for upholding the associated bundles of rights through, e.g., monitoring of properties), where GO indicates government organization. The bundles of rights associated with the tenure regimes are characterized according to past and current legislation in Brazil, with color shading from red to green indicating the extensiveness and/or level of guarantee of rights granted along seven different rights dimensions (access, subsistence withdrawal, commercial withdrawal, management, exclusion, alienation, due process).

| **Brazil tenure categories** | **Tenure regime** | **Tenants** | **Bundles of rights** (usually included) | | | | | | | **Main right holder** | **Main duty holder** | **References** |  |
| --- | --- | --- | --- | --- | --- | --- | --- | --- | --- | --- | --- | --- | --- |
|  |  |  | Access | Withdrawal (subsistence) | Withdrawal (commercial) | Management | Exclusion | Alienation | Due Process |  |  |  |  |
| CAR poor (properties with more than 5% of overlapping areas with neighbors) | Private lands | 1 | ++ | + | + | + | ++ | ++ | ++ | Individual(s), firm, or other  entity | Individual(s), firm, or other entity | Lei 4.947 art. 22 1966, ^87^ |  |
| CAR premium (properties with less than 5% of overlapping areas with neighbors) |  |  |  |  |  |  |  |  |  |  |  |  |  |
| SIGEF (Private properties registered in INCRA systems) |  |  |  |  |  |  |  |  |  |  |  |  |  |
| Private properties from Terra Legal program |  |  |  |  |  |  |  |  |  |  |  |  |  |
| Communitary lands | Communal lands | Many | ++ | + | +/- | +/- | +/- | +/- | - | Community | GO | Decreto N. 6.040, 2007, Lei N. 11.284, 2006, ^88,89^. |  |
| Quilombola lands | Quilombola lands | Usually many | ++ | + | + | + | + | -- | + | Community | Community | Consitucao Federal art. 68, Decreto N. 6.040, 2007, ^90,91^. |  |
| Homologated Indigenous land | Indigenous lands | Usually many | ++ | + | -- | + | + | -- | + | Community | GO | Consitucao Federal art. 231. 1996, Decreto N. 6.040, 2007, ^92^. |  |
| Non-homologated indigenous land |  |  |  |  |  |  |  |  |  |  |  |  |  |
| Full protection conservation unit | Strictly Protected Areas | 1 or few | - | -- | -- | + | ++ | -- | + | Citizenry | GO | Lei nº 6.938, de 31 de agosto de 1981, Lei Complementar n° 140, de 8 de dezembro de 2011 |  |
| Sustainable use conservation unit | Sustainable use Protected Areas | 1 or few | +/- | +/- | +/- | + | + | -- | + | Citizenry/  Community | GO | Lei nº 6.938, de 31 de agosto de 1981, Lei Complementar n° 140, de 8 de dezembro de 2011 |  |
| Rural settlements | Undesignated/untitled public lands | 1 or few | ++ | ++ | ++ | + | - | - | -- | Citizenry | GO | Lei Nº 12.465, de 11 de julho de 2017, ^9^ |  |
| Undesignated public forests |  |  |  |  |  |  |  |  |  |  |  |  |  |
| Undesignated lands from Terra legal program |  |  |  |  |  |  |  |  |  |  |  |  |  |
| Military areas, Water, and Urban | (omitted from analysis) | | | | | | | | | | | | |

++ indicates full guarantee of extensive rights
+ indicates some guaranteed rights that are usually subject to specific (e.g., environmental) restrictions
+/- indicates some rights, guaranteed under certain legal conditions, circumstances, or clauses
- indicates little guarantee of, or severely limited, rights
-- indicates no guarantee of any rights

Supplementary Table 3. Record of regression models for which federal states were merged into groups to facilitate the full estimation of parameter coefficients in GLMs in cases where insufficient observations across states prevented it. Geographically adjacent states were consecutively merged *(States grouped*). Model conversion was not achieved in 3/6 models (*Model convergence*), and are thus not reported in results (Tables S3-4).

| **Comparison** | **Temporal scale** | **Spatial scale** | **States grouped** | **States grouped** | **States grouped** | **Model convergence** |
| --- | --- | --- | --- | --- | --- | --- |
| Private vs. protected | 1985-2018 | Caatinga | SE+AL | PE+PB |  | no |
| Private vs. sustainable use | 1985-2018 | Caatinga | SE+AL | MG+BA |  | no |
| Private vs. communal | 1991-1995 | Brazil | SE+AL |  |  | yes |
| Private vs. sustainable use | 2013-2018 | Caatinga | SE+AL | PE+PB |  | no |
| Private vs. protected (PA filter) | 2013-2018 | Brazil | SE+AL | PB+RN | RR+TO | yes |
| Private vs. sustainable use (PA filter) | 1985-2018 | Brazil | MS+SP | AP+PA | PB+RN | yes |

**Supplementary Table 4.** Synthesis of the directions and relative magnitudes of effects of different land-tenure regimes across spatiotemporal scales. For this cross-scale synthesis, we considered all scales at which deforestation effects of all five alternative tenure regimes were consistently testable vis-à-vis the respective counterfactual (top part: undesignated/untitled; bottom part: private). The left section of the table (‘Direction of estimated effects on deforestation’) reports, for each tenure regime, the numbers and percentages of scale-specific model estimates predicting an increase or decrease in the likelihood of deforestation of all alternative tenure regimes vis-à-vis the counterfactual. The right section of the table (‘Ranking by relative magnitude of effect size’) reports the percentages of all compared spatiotemporal scales where each regime ranked as more deforestation-decreasing (‘best’) and less deforestation-decreasing/more increasing (‘worst’) than all alternatives regimes (based on their respective effect sizes). In this ranking, we placed effects that were statistically indistinguishable from 0 in between deforestation-decreasing and -increasing. For example, private land tenure reduced deforestation vis-à-vis an undesignated/untitled public regime more effectively (larger negative effect size) than all alternative regimes at 2.94% of the compared spatiotemporal scales, while decreasing deforestation least effectively or most strongly increasing deforestation at 25.49% of scales. We additionally report all percentages as weighted by the level of balance (*L_1_*) in the underlying dataset, which downweights cases where datasets still had low levels of overlap in covariate values post-matching. Note that in order to keep comparisons consistently comparable across spatiotemporal scales, this table does not include results for Pampa and Pantanal, nor comparisons against communal lands. Also note that these percentages synthesize ‘narrower scales’ only. For Brazil-wide results for the full 1985-2018 period, See Supplementary Figs 2-3.

|  | **Direction of estimated effects on deforestation** | | | | | | | | | | | | | | |  | **Ranking by relative magnitude of effect size** | | | | | | |
| --- | --- | --- | --- | --- | --- | --- | --- | --- | --- | --- | --- | --- | --- | --- | --- | --- | --- | --- | --- | --- | --- | --- | --- |
|  | increases (count) | increases (count) weighted by balance | | % increases | % increases weighted by balance | decreases (count) | decreases (count) weighted by balance | | % decreases | % decreases weighted by balance | non-significant (count) | non-significant weighted by balance | % non-significant | % non-significant weighted by balance | Total models | best | | best weighted by balance | worst | worst weighted by balance | non-significant | non-significant weighted by balance | Total models |
| **Compared to undesignated/untitled lands** | | | | | | | | | | | | | | | |  | |  |  |  |  |  |  |
| Private lands | 3 | 2.27 | 8.82% | | 8.38% | 23 | 18.34 | 67.65% | | 67.81% | 8 | 6.44 | 23.53% | 23.80% | 34 | 2.94% | | 2.22% | 26.23% | 28.03% | 8 | 2.66 | 34 |
| Protected areas | 0 | 0.00 | 0.00% | | 0.00% | 30 | 14.62 | 88.24% | | 90.19% | 4 | 1.59 | 11.76% | 9.81% | 34 | 26.47% | | 26.26% | 13.97% | 10.62% | 4 | 1.31 | 34 |
| Sustainable use areas | 0 | 0.00 | 0.00% | | 0.00% | 26 | 15.09 | 76.47% | | 79.07% | 8 | 3.99 | 23.53% | 20.93% | 34 | 41.18% | | 42.58% | 8.82% | 7.35% | 8 | 2.71 | 34 |
| Indigenous lands | 1 | 0.36 | 2.94% | | 2.32% | 26 | 12.97 | 76.47% | | 82.82% | 7 | 2.33 | 20.59% | 14.86% | 34 | 14.71% | | 17.82% | 12.99% | 10.45% | 7 | 2.33 | 34 |
| Quilombola lands | 1 | 0.49 | 2.94% | | 3.02% | 17 | 7.85 | 50.00% | | 48.69% | 16 | 7.79 | 47.06% | 48.28% | 34 | 14.71% | | 11.12% | 37.99% | 43.55% | 16 | 6.50 | 34 |
| *All of the above compared to undesignated/untitled* | **4** | **3.12** | **2.35%** | | **3.31%** | **122** | **68.87** | **71.76%** | | **73.17%** | **43** | **22.13** | **25.29%** | **23.52%** | **170** |  | |  |  |  |  |  |  |
| **Compared to private lands** | | | | | | | | | | | | | | | |  | |  |  |  |  |  |  |
| Public lands | 21 | 16.61 | 77.78% | | 79.09% | 3 | 2.27 | 11.11% | | 10.79% | 3 | 2.13 | 11.11% | 10.12% | 27 | 0.00% | | 0.00% | 81.48% | 85.70% | 3 | 0.86 | 27 |
| Protected areas | 0 | 0.00 | 0.00% | | 0.00% | 21 | 12.70 | 77.78% | | 83.60% | 6 | 2.49 | 22.22% | 16.40% | 27 | 11.11% | | 8.64% | 6.17% | 4.55% | 6 | 1.89 | 27 |
| Sustainable use areas | 0 | 0.00 | 0.00% | | 0.00% | 24 | 14.43 | 88.89% | | 90.40% | 3 | 1.53 | 11.11% | 9.60% | 27 | 44.44% | | 52.20% | 3.09% | 1.53% | 3 | 0.72 | 27 |
| Indigenous lands | 0 | 0.00 | 0.00% | | 0.00% | 19 | 8.10 | 70.37% | | 59.39% | 8 | 5.54 | 29.63% | 40.61% | 27 | 40.74% | | 36.19% | 0.00% | 0.00% | 8 | 5.18 | 27 |
| Quilombola lands | 0 | 0.00 | 0.00% | | 0.00% | 11 | 6.39 | 40.74% | | 40.11% | 16 | 9.53 | 59.26% | 59.89% | 27 | 10.48% | | 8.63% | 10.48% | 9.56% | 21 | 9.26 | 31 |
| *All of the above compared to private* | **21** | **16.61** | **15.56%** | | **20.33%** | **78** | **43.88** | **57.78%** | | **53.70%** | **36** | **21.22** | **26.67%** | **25.97%** | **135** |  | |  |  |  |  |  |  |

**Supplementary Table 5.** Synthesized direction of cross-scale effects of different land-tenure regimes, but focusing on scales remaining after time-filtering strict-protection and sustainable-use regimes, with percentages based on alternative results that were time-filtered for greater robustness of temporal stability assumptions (see sections 2.2. and 3.5; see Supplementary Table 6 for detailed description). These time-filtered datasets exclude any parcels for which these respective conservation-focused tenure regime was either not yet established at the beginning of the considered time period or for which the creation date was unknown. Note that in left first table section (‘Direction of estimated effects on deforestation’), only the results for strict-protection and sustainable-use regimes (in black) are based on different models. Those for other tenure regimes are as in Supplementary Table 5, but restricted to the scales where all regimes could be consistently compared. We note that due to smaller initial parcel numbers of the time-filtered datasets, the matched time-filtered datasets showed substantially lower balance levels post-matching compared to the non-filtered datasets (see Source Data file). Therefore, we do not consider the ranking results (‘Ranking by relative magnitude of effect size’) based on the time-filtered data reliable, and ignored them in our conclusions. They are shown here (in grey) for transparency only.

|  | **Direction of estimated effects on deforestation** | | | | | | | | | | | | | | | **Ranking by relative magnitude of effect size** | | | | | | |
| --- | --- | --- | --- | --- | --- | --- | --- | --- | --- | --- | --- | --- | --- | --- | --- | --- | --- | --- | --- | --- | --- | --- |
|  | increases (count) | increases (count) weighted by balance | % increases | % increases weighted by balance | decreases (count) | decreases (count) weighted by balance | % decreases | % decreases weighted by balance | non-significant (count) | | non-significant weighted by balance | | % non-significant | % non-significant weighted by balance | Total models | best | best weighted by balance | worst | worst weighted by balance | non-significant | non-significant weighted by balance | Total models |
| **Compared to undesignated/untitled lands** | | | | | | | | | | | | | | | |  |  |  |  |  |  |  |
| Private lands | 1 | 0.88 | 5.26% | 5.70% | 17 | 13.65 | 89.47% | 88.68% | 1 | 0.87 | | 5.26% | | 5.62% | 19 | 5.26% | 3.48% | 27.63% | 30.69% | 1 | 0 | 19 |
| Protected areas | 0 | 0.00 | 0.00% | 0.00% | 18 | 9.63 | 94.74% | 96.12% | 1 | 0.39 | | 5.26% | | 3.88% | 19 | 15.79% | 12.51% | 7.89% | 5.22% | 1 | 0 | 19 |
| Sustainable use areas | 0 | 0.00 | 0.00% | 0.00% | 15 | 8.51 | 78.95% | 80.55% | 4 | 2.06 | | 21.05% | | 19.45% | 19 | 47.37% | 52.65% | 9.21% | 6.60% | 4 | 1 | 19 |
| Indigenous lands | 0 | 0.00 | 0.00% | 0.00% | 18 | 9.71 | 94.74% | 96.54% | 1 | 0.35 | | 5.26% | | 3.46% | 19 | 26.32% | 27.89% | 11.84% | 7.93% | 1 | 0 | 19 |
| Quilombola lands | 0 | 0.00 | 0.00% | 0.00% | 12 | 5.87 | 63.16% | 61.53% | 7 | 3.67 | | 36.84% | | 38.47% | 19 | 5.26% | 3.48% | 43.42% | 49.57% | 7 | 3 | 19 |
| *All of the above compared to undesignated/untitled* | 5 | 0.88 | 5.26% | 1.58% | 80 | 47.38 | 84.21% | 85.24% | 14 | 7.33 | | 14.74% | | 13.19% | 95 |  |  |  |  |  |  |  |
| **Compared to private lands** | | | | | | | | | | | | | | | |  |  |  |  |  |  |  |
| Public lands | 16 | 13.00 | 94.12% | 93.68% | 1 | 0.88 | 5.88% | 6.32% | 0 | 0.00 | | 0.00% | | 0.00% | 17 | 0.00% | 0.00% | 94.12% | 93.05% | 0 | 0 | 17 |
| Protected areas | 0 | 0.00 | 0.00% | 0.00% | 15 | 9.52 | 88.24% | 89.19% | 2 | 1.15 | | 11.76% | | 10.81% | 17 | 17.65% | 18.28% | 0.00% | 0.00% | 2 | 1 | 17 |
| Sustainable use areas | 0 | 0.00 | 0.00% | 0.00% | 13 | 8.34 | 76.47% | 74.87% | 4 | 2.80 | | 23.53% | | 25.13% | 17 | 35.29% | 38.65% | 2.94% | 3.47% | 4 | 2 | 17 |
| Indigenous lands | 0 | 0.00 | 0.00% | 0.00% | 10 | 5.25 | 58.82% | 52.39% | 7 | 4.77 | | 41.18% | | 47.61% | 17 | 41.18% | 39.21% | 0.00% | 0.00% | 7 | 4 | 17 |
| Quilombola lands | 0 | 0.00 | 0.00% | 0.00% | 7 | 4.48 | 41.18% | 39.52% | 10 | 6.86 | | 58.82% | | 60.48% | 17 | 5.88% | 3.85% | 2.94% | 3.47% | 10 | 6 | 17 |
| *All of the above compared to private* | 16 | 13.00 | 18.82% | 22.79% | 46 | 28.47 | 54.12% | 49.89% | 23 | 15.59 | | 27.06% | | 27.31% | 85 |  |  |  |  |  |  |  |

Supplementary Table 6. Synthesized direction of effects of all assessed land-tenure regimes on deforestation across all assessed scales (see Supplementary Tables 5-6 for general description). Unlike results in Tables 5-6, which consider only tenure regimes and scales for which consistent comparisons were possible, results here are based on all ‘narrower’ scales where a given land-tenure regime could be compared against the respective counterfactual (i.e., excl. results for Brazil for the 1985-2018 period, but also incl., e.g., private-vs-undesignated/untitled comparisons for Pampa and Pantanal). These results are thus more comprehensive (based on more scales) than those in Tables 5-6 if single tenure regimes are viewed in isolation. However, unlike results in Tables 5-6, they are not comparable across tenure regimes as they are based on inconsistent combinations of scales. Information that is redundant with that in Supplementary Table 5 (as based on the same scales) is shown in grey.

|  | **Direction of estimated effects on deforestation** | | | | | | | | | | | | | | |
| --- | --- | --- | --- | --- | --- | --- | --- | --- | --- | --- | --- | --- | --- | --- | --- |
|  | increases (count) | increases (count) weighted by balance | % increases | % increases weighted by balance | decreases (count) | decreases (count) weighted by balance | | % decreases | % decreases weighted by balance | non-significant (count) | non-significant weighted by balance | % non-significant | % non-significant weighted by balance | Total models |  |
| **Compared to undesignated/untitled lands** | | | | | | | | | | | | | | | |
| Private lands | 4 | 2.81 | 8.70% | 8.33% | 30 | 22.18 | 65.22% | | 65.83% | 13 | 9.25 | 27.66% | 27.02% | 47 |  |
| Protected areas | 0 | 0.00 | 0.00% | 0.00% | 30 | 14.62 | 88.24% | | 90.19% | 4 | 1.59 | 11.76% | 9.81% | 34 |  |
| Sustainable use areas | 0 | 0.00 | 0.00% | 0.00% | 26 | 15.09 | 76.47% | | 79.07% | 8 | 3.99 | 23.53% | 20.93% | 34 |  |
| Indigenous lands | 1 | 0.36 | 2.94% | 2.32% | 26 | 12.97 | 76.47% | | 82.82% | 7 | 2.33 | 20.59% | 14.86% | 34 |  |
| Quilombola lands | 1 | 0.49 | 2.94% | 3.02% | 17 | 7.85 | 50.00% | | 48.69% | 16 | 7.79 | 47.06% | 48.28% | 34 |  |
| Communal lands | 0 | 0.00 | 0.00% | 0.00% | 12 | 7.78 | 92.31% | | 91.22% | 1 | 0.75 | 7.69% | 8.78% | 13 |  |
| *All of the above compared to undesignated/untitled* | 6 | 3.66 | 3.08% | 3.35% | 141 | 80.48 | 72.31% | | 73.64% | 49 | 25.70 | 25.00% | 23.39% | 196 |  |
| Robustness check: protected areas and sustainable use areas filtered by known year of creation | | | | | | | | | | | | | | | |
| Protected areas | 0 | 0 | 0.00% | 0.00% | 23 | 11.60 | 88.46% | | 91.10% | 3 | 1.13 | 11.54% | 8.90% | 26 |  |
| Sustainable use areas | 0 | 0 | 0.00% | 0.00% | 15 | 8.51 | 78.95% | | 80.55% | 4 | 2.06 | 21.05% | 19.45% | 19 |  |
| *All of the above compared to undesignated/untitled  (using filtered results instead)* | 6 | 3.66 | 3.49% | 3.76% | 123 | 70.89 | 71.51% | | 72.85% | 44 | 23.30 | 25.43% | 23.81% | 173 |  |
| **Compared to private lands** |  |  |  |  |  |  |  | |  |  |  |  |  |  |  |
| Public lands | 29 | 21.64 | 61.70% | 63.22% | 4 | 2.81 | 8.51% | | 8.20% | 14 | 9.79 | 29.79% | 28.58% | 47 |  |
| Protected areas | 0 | 0.00 | 0.00% | 0.00% | 25 | 15.69 | 75.76% | | 81.21% | 8 | 3.63 | 24.24% | 18.79% | 33 |  |
| Sustainable use areas | 0 | 0.00 | 0.00% | 0.00% | 27 | 16.42 | 84.38% | | 84.50% | 5 | 3.01 | 15.63% | 15.50% | 32 |  |
| Indigenous lands | 0 | 0.00 | 0.00% | 0.00% | 19 | 8.10 | 67.86% | | 57.97% | 9 | 5.87 | 32.14% | 42.03% | 28 |  |
| Quilombola lands | 1 | 0.69 | 2.94% | 3.35% | 11 | 6.39 | 32.35% | | 30.86% | 22 | 13.61 | 64.71% | 65.79% | 34 |  |
| Communal lands | 1 | 0.35 | 7.69% | 7.16% | 9 | 3.48 | 69.23% | | 70.34% | 3 | 1.11 | 23.08% | 22.50% | 13 |  |
| *All of the above compared to private* | 31 | 22.69 | 16.58% | 20.15% | 95 | 52.88 | 50.80% | | 46.96% | 61 | 37.03 | 32.62% | 32.88% | 187 |  |
| Robustness check: protected areas and sustainable use areas filtered by known year of creation | | | | | | | | | | | | | | | |
| Protected areas | 0 | 0.00 | 0.00% | 0.00% | 20 | 12.41 | 83.33% | | 86.03% | 4 | 2.01 | 16.67% | 13.97% | 24 |  |
| Sustainable use areas | 0 | 0.00 | 0.00% | 0.00% | 14 | 8.73 | 77.78% | | 75.73% | 4 | 2.80 | 22.22% | 24.27% | 18 |  |
| *All of the above compared to private  (using filtered results instead)* | 31 | 22.69 | 18.90% | 22.74% | 77 | 41.91 | 46.95% | | 42.00% | 56 | 35.20 | 34.15% | 35.27% | 164 |  |

**Supplementary Table 7.** Summary of sensitivity analysis using Rosenbaum bounds. We calculate upper and lower bounds for both Hodges Lehmann point estimates and *p*-values (see supplementary file #2 for full results) for different Γ levels. For each tenure-regime comparison, spatial scale, and temporal scale considered, we summarize *i*) the geometric mean deviation of upper/lower bounds of Hodges Lehmann estimates from Γ=1, with deviations expressed as relative error in percent (i.e., relative to the magnitude of the respective median effect size at Γ=1), and *ii*) the percent of models that changed in statistical significance (*p*≤0.05). See Supplementary Data 3 for full results.

|  | **Geometric mean deviation of upper/lower bounds of Hodges Lehmann estimates from Γ=1 (deviation expressed as relative error in percent)** | | | | | **Percentage of models that change in significance (p≤0.05) from Γ=1** | | | | |
| --- | --- | --- | --- | --- | --- | --- | --- | --- | --- | --- |
| **Tenure-regime comparisons** | Γ =1.1 | Γ =1.2 | Γ =1.3 | Γ =1.4 | Γ =1.5 | Γ =1.1 | Γ =1.2 | Γ =1.3 | Γ =1.4 | Γ =1.5 |
| public vs. private | 12.54% | 23.20% | 31.57% | 41.62% | 49.25% | 6.25% | 10.42% | 12.50% | 14.58% | 20.83% |
| public vs. protected | 8.05% | 15.97% | 22.73% | 28.43% | 34.59% | 5.71% | 5.71% | 5.71% | 8.57% | 8.57% |
| public vs. sustainable use | 6.69% | 13.00% | 18.38% | 23.87% | 29.16% | 2.86% | 5.71% | 17.14% | 17.14% | 28.57% |
| public vs. indigenous | 12.72% | 23.31% | 32.44% | 36.92% | 45.80% | 0.00% | 11.43% | 11.43% | 11.43% | 14.29% |
| public vs. quilombola | 21.39% | 37.18% | 56.50% | 72.26% | 83.73% | 2.86% | 11.43% | 14.29% | 20.00% | 20.00% |
| public vs. communal | 8.14% | 15.81% | 22.96% | 29.59% | 35.79% | 0.00% | 0.00% | 0.00% | 7.69% | 7.69% |
| private vs. public | 11.10% | 20.95% | 26.96% | 37.62% | 44.59% | 6.25% | 10.42% | 12.50% | 14.58% | 18.75% |
| private vs. protected | 8.56% | 16.81% | 24.82% | 32.04% | 38.54% | 2.94% | 8.82% | 11.76% | 17.65% | 17.65% |
| private vs. sustainable use | 7.68% | 15.10% | 21.92% | 28.40% | 34.57% | 0.00% | 3.03% | 3.03% | 12.12% | 21.21% |
| private vs. indigenous | 11.78% | 22.68% | 31.45% | 37.76% | 45.25% | 3.45% | 6.90% | 6.90% | 20.69% | 24.14% |
| private vs. quilombola | 26.46% | 47.77% | 66.12% | 80.85% | 103.00% | 5.71% | 11.43% | 14.29% | 22.86% | 28.57% |
| private vs. communal | 9.11% | 17.51% | 25.34% | 32.62% | 39.52% | 0.00% | 0.00% | 0.00% | 0.00% | 8.33% |
| *Average across tenure-regime comparisons* | *12.02%* | *22.44%* | *31.77%* | *40.17%* | *48.65%* | *3.00%* | *7.11%* | *9.13%* | *13.94%* | *18.22%* |
| **Spatial scales** |  |  |  |  |  |  |  |  |  |  |
| Brazil | 9.91% | 18.51% | 24.03% | 32.31% | 40.12% | 1.23% | 2.47% | 2.47% | 7.41% | 13.58% |
| Amazonia | 12.97% | 24.72% | 35.44% | 44.84% | 54.36% | 5.95% | 10.71% | 16.67% | 21.43% | 28.57% |
| Caatinga | 21.45% | 41.81% | 60.40% | 79.84% | 95.11% | 4.92% | 13.11% | 18.03% | 22.95% | 26.23% |
| Cerrado | 12.14% | 22.89% | 31.19% | 35.93% | 42.24% | 1.43% | 11.43% | 11.43% | 15.71% | 18.57% |
| Mata Atlantica | 7.41% | 13.56% | 20.28% | 26.32% | 31.93% | 2.86% | 4.29% | 5.71% | 11.43% | 12.86% |
| Pampa | 4.93% | 9.39% | 13.56% | 17.37% | 20.98% | 0.00% | 0.00% | 0.00% | 0.00% | 0.00% |
| Pantanal | 9.91% | 19.25% | 27.97% | 35.57% | 42.71% | 16.67% | 16.67% | 16.67% | 16.67% | 25.00% |
| *Average across spatial scales* | *11.25%* | *21.45%* | *30.41%* | *38.88%* | *46.78%* | *4.72%* | *8.38%* | *10.14%* | *13.66%* | *17.83%* |
| **Temporal scales** |  |  |  |  |  |  |  |  |  |  |
| 1985-2018 | 8.67% | 16.52% | 23.55% | 29.83% | 35.58% | 1.79% | 8.93% | 8.93% | 12.50% | 17.86% |
| 1985-1990 | 15.47% | 28.23% | 33.11% | 41.12% | 52.36% | 3.64% | 9.09% | 14.55% | 18.18% | 25.45% |
| 1991-1995 | 16.40% | 32.42% | 45.49% | 56.32% | 66.32% | 5.36% | 8.93% | 8.93% | 12.50% | 19.64% |
| 1996-1999 | 10.71% | 20.62% | 29.82% | 38.97% | 47.27% | 1.75% | 8.77% | 10.53% | 14.04% | 17.54% |
| 2000-2004 | 10.41% | 18.90% | 28.46% | 36.88% | 44.65% | 1.79% | 5.36% | 7.14% | 14.29% | 16.07% |
| 2005-2012 | 10.95% | 21.22% | 30.91% | 39.44% | 47.09% | 1.79% | 5.36% | 10.71% | 16.07% | 19.64% |
| 2013-2018 | 9.19% | 16.90% | 24.43% | 31.80% | 38.10% | 8.93% | 10.71% | 12.50% | 17.86% | 19.64% |
| *Average across spatial scales* | *11.69%* | *22.12%* | *30.82%* | *39.19%* | *47.34%* | *3.58%* | *8.16%* | *10.47%* | *15.06%* | *19.41%* |

**Supplementary Table 8.** Summary of mean differences in key covariates between matched sample and entire population of Brazilian parcels. For each covariate (elevation (in meters), slope (in degrees), travel time to nearest city (in minutes), human population density , and area (in ha)), we compare the means of both matched sample and the entire population (based on a stratified representative sample of parcels), and report the larger values in bold, for visual aid (e.g. for public vs. private on average, the matched sample had lower elevation than the entire population). We also report the absolute standardized mean difference (ASMD) between the matched sample and the population as a measure of these differences, with values closer to 0 indicating no differences between groups.

| **Tenure-regime comparison** | Mean elevation (matched) | Mean elevation (population) | Elevation ASMD | Mean slope (matched) | Mean slope (matched) | Slope ASMD | Mean travel time (matched) | Mean travel time (population) | Travel time ASMD | Mean population (matched) | Mean population (population) | Human population ASMD | Mean area (matched) | Mean area (population) | Area ASMD |
| --- | --- | --- | --- | --- | --- | --- | --- | --- | --- | --- | --- | --- | --- | --- | --- |
| Public vs. private | 272.113 | **374.451** | 0.451 | 1.056 | **1.804** | 0.537 | **215.834** | 170.089 | 0.260 | 0.949 | **1.460** | 0.087 | **1594.247** | 342.993 | 0.460 |
| Public vs. protected | **376.100** | 313.589 | 0.349 | **1.244** | 1.208 | 0.226 | **458.325** | 262.197 | 0.414 | **1.611** | 1.582 | 0.098 | **68175.168** | 4247.486 | 0.507 |
| Public vs. sustainable_use | **323.718** | 312.660 | 0.296 | **1.468** | 1.193 | 0.239 | **382.016** | 252.794 | 0.374 | **1.483** | 1.405 | 0.106 | **48957.248** | 4548.720 | 0.316 |
| Public vs. indigenous | 266.058 | **311.891** | 0.307 | 0.989 | **1.205** | 0.246 | **519.122** | 264.588 | 0.345 | **1.236** | 1.202 | 0.128 | **37307.475** | 9306.220 | 0.436 |
| Public vs. quilombola | 214.454 | **315.179** | 0.704 | 1.064 | **1.142** | 0.253 | 199.802 | **260.053** | 0.294 | **1.333** | 1.229 | 0.141 | **6424.032** | 3900.827 | 0.284 |
| Public vs. communal | 69.436 | **220.601** | 0.989 | **0.130** | 0.769 | 0.784 | **555.164** | 396.144 | 0.320 | 0.649 | **0.881** | 0.056 | **3459.764** | 10141.409 | 0.113 |
| Private vs. public | 272.113 | **374.650** | 0.456 | 1.056 | **1.816** | 0.544 | **215.834** | 169.831 | 0.264 | 0.949 | **1.493** | 0.090 | **1594.247** | 337.529 | 0.455 |
| Private vs. protected | 423.387 | **452.292** | 0.278 | 1.798 | **2.039** | 0.214 | **370.432** | 179.991 | 0.342 | **4.044** | 1.706 | 0.190 | **27280.312** | 106.919 | 0.656 |
| Private vs. sustainable use | **451.900** | 450.391 | 0.237 | **2.773** | 2.021 | 0.383 | **290.159** | 181.754 | 0.273 | **2.230** | 1.749 | 0.096 | **15131.491** | 119.635 | 0.398 |
| Private vs. indigenous | 280.292 | **456.633** | 0.797 | 1.273 | **2.048** | 0.572 | **463.940** | 186.798 | 0.611 | 1.520 | **1.772** | 0.151 | **16857.519** | 407.279 | 0.707 |
| Private vs. quilombola | 248.856 | **456.667** | 1.021 | 1.370 | **2.024** | 0.501 | **207.032** | 184.176 | 0.177 | **2.805** | 1.741 | 0.108 | **6073.282** | 126.352 | 0.504 |
| Private vs. communal | 58.595 | **334.289** | 1.641 | 0.293 | **1.701** | 1.141 | **532.994** | 255.234 | 0.673 | 0.843 | **1.458** | 0.090 | **1529.501** | 189.499 | 0.077 |
| **Spatial Scale** |  |  |  |  |  |  |  |  |  |  |  |  |  |  |  |
| Brazil | 269.445 | **415.363** | 0.652 | 1.347 | **1.859** | 0.478 | **380.721** | 192.179 | 0.434 | **1.826** | 1.631 | 0.094 | **18756.604** | 2309.234 | 0.245 |
| Amazonia | 95.032 | **159.394** | 0.698 | 0.384 | **0.776** | 0.566 | **821.159** | 439.857 | 0.544 | 0.594 | **0.869** | 0.082 | **67660.044** | 7661.397 | 0.444 |
| Caatinga | 331.144 | **380.221** | 0.394 | **1.458** | 1.360 | 0.340 | 136.230 | **137.341** | 0.167 | **2.064** | 1.901 | 0.140 | **3544.037** | 338.386 | 0.557 |
| Cerrado | 486.524 | **539.708** | 0.702 | 1.189 | **1.495** | 0.393 | **204.386** | 188.665 | 0.295 | **1.345** | 1.127 | 0.156 | **5553.383** | 669.125 | 0.468 |
| Mata Atlantica | 375.530 | **494.644** | 0.425 | 2.565 | **2.951** | 0.259 | **111.219** | 109.910 | 0.256 | **3.368** | 2.414 | 0.099 | **1231.630** | 313.810 | 0.469 |
| Pampa | **177.034** | 171.840 | 0.055 | 0.856 | **1.685** | 0.797 | **113.198** | 94.173 | 0.322 | 0.226 | **1.001** | 0.208 | **920.966** | 76.675 | 1.267 |
| Pantanal | 182.032 | **182.671** | 0.054 | 0.523 | **0.855** | 0.298 | 169.578 | **182.573** | 0.087 | 0.255 | **0.492** | 0.074 | **1302.871** | 1577.547 | 0.059 |
| **Temporal Scale** |  |  |  |  |  |  |  |  |  |  |  |  |  |  |  |
| 1985-2018 | 292.993 | **376.770** | 0.532 | 1.301 | **1.641** | 0.435 | **336.681** | 215.714 | 0.340 | **1.774** | 1.422 | 0.120 | **19792.971** | 2434.078 | 0.458 |
| 1985-1990 | 292.712 | **372.965** | 0.553 | 1.300 | **1.614** | 0.431 | **336.054** | 212.098 | 0.340 | **1.370** | 1.360 | 0.114 | **19731.498** | 1881.821 | 0.446 |
| 1991-1995 | 292.996 | **369.200** | 0.533 | 1.302 | **1.603** | 0.422 | **336.169** | 213.175 | 0.347 | **1.439** | 1.420 | 0.103 | **19738.298** | 2768.898 | 0.443 |
| 1996-1999 | 293.252 | **376.679** | 0.545 | 1.301 | **1.621** | 0.412 | **336.625** | 216.628 | 0.336 | **1.577** | 1.358 | 0.124 | **19792.447** | 2161.936 | 0.443 |
| 2000-2004 | 293.204 | **373.309** | 0.532 | 1.301 | **1.673** | 0.431 | **336.627** | 219.472 | 0.346 | **1.776** | 1.520 | 0.122 | **19795.835** | 2588.518 | 0.443 |
| 2005-2012 | 293.597 | **378.865** | 0.560 | 1.305 | **1.652** | 0.421 | **340.257** | 216.207 | 0.344 | **1.873** | 1.646 | 0.115 | **20049.582** | 2734.127 | 0.443 |
| 2013-2018 | 291.141 | **374.460** | 0.566 | 1.317 | **1.646** | 0.426 | **341.225** | 218.240 | 0.336 | **1.999** | 1.830 | 0.100 | **20077.450** | 1956.230 | 0.441 |

**Supplementary References**

1. Damasceno, R., Chiavari, J. & Leme Lopes, C. *Evolution of land rights in rural Brazil*. https://climatepolicyinitiative.org/wp-content/uploads/2017/06/Evolution_of_Land_Rights_In_Rural_Brazil_CPI_FinalEN.pdf (2017).

2. World Bank. *Brazil - Land governance assessment*. http://documents.worldbank.org/curated/en/105561468191049199/Brazil-Land-governance-assessment (2014).

3. Fearnside, P. M. Land-Tenure Issues as Factors in Environmental Destruction in Brazilian Amazonia: The Case of Southern Pará. *World Development* **29**, 1361–1372 (2001).

4. Cunha, F. A. F. de S., Börner, J., Wunder, S., Cosenza, C. A. N. & Lucena, A. F. P. The implementation costs of forest conservation policies in Brazil. *Ecological Economics* **130**, 209–220 (2016).

5. Soterroni, A. C. *et al.* Expanding the Soy Moratorium to Brazil’s Cerrado. *Science Advances* **5**, eaav7336 (2019).

6. Soares-Filho, B. *et al.* Cracking Brazil’s Forest Code. *Science* **344**, 363–364 (2014).

7. Azevedo, A. A. *et al.* Limits of Brazil’s Forest Code as a means to end illegal deforestation. *PNAS* **114**, 7653–7658 (2017).

8. Imaflora, GeoLab (ESALQ/USP), Royal Institute of Technology in Stockholm (KHT) & Instituto Federal de Educação, Ciência e Tecnologia de São Paulo (IF/SP). Atlas - The geography of Brazilian agriculture. (2018).

9. Sparovek, G. *et al.* Who owns Brazilian lands? *Land Use Policy* **87**, 104062 (2019).

10. Robinson, B. E. *et al.* Incorporating Land Tenure Security into Conservation: Conservation and land tenure security. *Conservation Letters* **11**, e12383 (2017).

11. Duchelle, A. E. *et al.* Linking Forest Tenure Reform, Environmental Compliance, and Incentives: Lessons from REDD+ Initiatives in the Brazilian Amazon. *World Development* **55**, 53–67 (2014).

12. Probst, B., BenYishay, A., Kontoleon, A. & dos Reis, T. N. P. Impacts of a large-scale titling initiative on deforestation in the Brazilian Amazon. *Nat Sustain* (2020) doi:10.1038/s41893-020-0537-2.

13. Ministerio do Meio Ambiente. *CNUC 2020 2ndo semestre*. https://dados.gov.br/dataset/unidadesdeconservacao/resource/c0babb3e-ec4e-4db5-a2b6-b79477260b0f?inner_span=True (2020).

14. Bowen, M. L. The struggle for black land rights in Brazil: an insider’s view on *quilombos* and the *quilombo* land movement. *African and Black Diaspora: An International Journal* **3**, 147–168 (2010).

15. *Project MapBiomas - Collection 4.0 of Brazilian Land Cover & Use Map Series*. mapbiomas.org.

16. Nelson, A. Travel time to major cities: A global map of Accessibility. *Office for Official Publications of the European Communities, Luxembourg* (2008) doi:10.2788/95835.

17. Yamazaki, D. *et al.* A high-accuracy map of global terrain elevations. *Geophysical Research Letters* **44**, 5844–5853 (2017).

18. Freire, S., Doxsey-Whitfield, E., MacManus, K., Mills, J. & Pesaresi, M. Development of new open and free multi-temporal global population grids at 250 m resolution. 7 (2016).

19. Joppa, L. N. & Pfaff, A. High and Far: Biases in the Location of Protected Areas. *PLOS ONE* **4**, e8273 (2009).

20. Bravo, K. Balancing Indigenous Rights to Land and the Demands of Economic Development: Lessons from the United States and Australia. *Columbia Journal of Law and Social Problems* **30**, 529–586 (1997).

21. Brown, T. Contestation, confusion and corruption: Market-based land reform in Zambia. in *Competing Jurisdictions* (eds. Evers, S., Spierenburg, M. & Wels, H.) 79–102 (BRILL, 2005). doi:10.1163/9789047416449_007.

22. Jakus, P. M. *et al.* Western Public Lands and the Fiscal Implications of a Transfer to States. *Land Economics* **93**, 371–389 (2017).

23. Burchfield, M., Overman, H. G., Puga, D. & Turner, M. A. Causes of Sprawl: A Portrait from Space. *The Quarterly Journal of Economics* **121**, 587–633 (2006).

24. Stefanes, M. *et al.* Property size drives differences in forest code compliance in the Brazilian Cerrado. *Land Use Policy* **75**, 43–49 (2018).

25. Iacus, S. M., King, G. & Porro, G. Causal Inference without Balance Checking: Coarsened Exact Matching. *Polit. anal.* **20**, 1–24 (2011).

26. Ferrante, L. & Fearnside, P. M. Brazil threatens Indigenous lands. *Science* **368**, 481–482 (2020).

27. Brito, B., Barreto, P., Brandão, A., Baima, S. & Gomes, P. H. Stimulus for land grabbing and deforestation in the Brazilian Amazon. *Environ. Res. Lett.* **14**, 064018 (2019).

28. Tollefson, J. Stopping deforestation: Battle for the Amazon. *Nature News* **520**, 20 (2015).

29. Shankland, A. & Gonçalves, E. Imagining Agricultural Development in South–South Cooperation: The Contestation and Transformation of ProSAVANA. *World Development* **81**, 35–46 (2016).

30. Meyfroidt, P. *et al.* Middle-range theories of land system change. *Global Environmental Change* **53**, 52–67 (2018).

31. Nepstad, D. *et al.* Slowing Amazon deforestation through public policy and interventions in beef and soy supply chains. *Science* **344**, 1118–1123 (2014).

32. Moutinho, P. *et al.* The emerging REDD+ regime of Brazil. *Carbon Management* **2**, 587–602 (2011).

33. Iacus, S. M., King, G. & Porro, G. A Theory of Statistical Inference for Matching Methods in Causal Research. *Polit. Anal.* **27**, 46–68 (2019).

34. Herrera, D., Pfaff, A. & Robalino, J. Impacts of protected areas vary with the level of government: Comparing avoided deforestation across agencies in the Brazilian Amazon. *Proc Natl Acad Sci USA* **116**, 14916–14925 (2019).

35. VanderWeele, T. J. Principles of confounder selection. *Eur J Epidemiol* **34**, 211–219 (2019).

36. Iacus, S. M., King, G. & Porro, G. cem: Software for Coarsened Exact Matching. *J. Stat. Soft.* **30**, (2009).

37. R Core Team. *R: A language and environment for statistical computing*. (R Foundation for Statistical Computing, 2020).

38. Vittinghoff, E. & McCulloch, C. E. Relaxing the Rule of Ten Events per Variable in Logistic and Cox Regression. *Am J Epidemiol* **165**, 710–718 (2007).

39. Greifer, N. & Stuart, E. A. Choosing the Estimand When Matching or Weighting in Observational Studies. *arXiv:2106.10577 [stat]* (2021).

40. Ackerman, B. *et al.* Implementing statistical methods for generalizing randomized trial findings to a target population. *Addictive Behaviors* **94**, 124–132 (2019).

41. Leeper, T. J. margins: Marginal Effects for Model Objects. (2021).

42. Leeper, T. J. Interpreting Regression Results using Average Marginal Eﬀects with R’s margins. (2017).

43. Albert, A. & Anderson, J. A. On the Existence of Maximum Likelihood Estimates in Logistic Regression Models. *Biometrika* **71**, 1–10 (1984).

44. Allison, P. Convergence Problems in Logistic Regression. in *Numerical Issues in Statistical Computing for the Social Scientist* 238–252 (John Wiley & Sons, Ltd, 2003). doi:10.1002/0471475769.ch10.

45. Pacheco, Andrea & Meyer, Carsten. Supplementary data for ‘Land tenure drives Brazil’s deforestation rates across socio-environmental contexts’. (2022) doi:10.5281/ZENODO.7068678.

46. Rosenbaum, P. R. Sensitivity Analysis for m-Estimates, Tests, and Confidence Intervals in Matched Observational Studies. *Biometrics* **63**, 456–464 (2007).

47. *Introduction to meta-analysis*. (John Wiley & Sons, 2009).

48. Pfaff, A., Robalino, J., Sandoval, C. & Herrera, D. Protected area types, strategies and impacts in Brazil’s Amazon: public protected area strategies do not yield a consistent ranking of protected area types by impact. *Phil. Trans. R. Soc. B* **370**, 20140273 (2015).

49. Gordon, H. S. The Economic Theory of a Common-Property Resource: The Fishery. *The Journal of Political Economy* **62**, 124–142 (1954).

50. Hardin, G. The Tragedy of the Commons. *Science* **162**, 1243–1248 (1968).

51. Browder, J. O., Godfrey, B. J. & Godfrey, B. *Rainforest cities: Urbanization, development, and globalization of the Brazilian Amazon*. (Columbia University Press, 1997).

52. Grafton, R. Q. Governance of the Commons: A Role for the State. *Land Economics* **76**, 504–517 (2000).

53. Sandler, T. Collective action: fifty years later. *Public Choice* **164**, 195–216 (2015).

54. de Soto, H. *The mystery of capital: Why capitalism triumphs in the West and fails everywhere else*. (Civitas Books, 2000).

55. Place, F. & Otsuka, K. Land Tenure Systems and Their Impacts on Agricultural Investments and Productivity in Uganda. *Journal of Development Studies* **38**, 105–128 (2002).

56. Deininger, K., Zegarra, E. & Lavadenz, I. Determinants and impacts of rural land market activity: Evidence from Nicaragua. *World Development* **31**, 1385–1404 (2003).

57. Binswanger, H. P. Brazilian policies that encourage deforestation in the Amazon. *World Development* **19**, 821–829 (1991).

58. Anderson, C. M., Asner, G. P., Llactayo, W. & Lambin, E. F. Overlapping land allocations reduce deforestation in Peru. *Land Use Policy* **79**, 174–178 (2018).

59. Liscow, Z. D. Do property rights promote investment but cause deforestation? Quasi-experimental evidence from Nicaragua. *Journal of Environmental Economics and Management* **65**, 241–261 (2013).

60. Perrings, C. An optimal path to extinction? Poverty and resource degradation in the open agrarian economy. *Journal of Development Economics* 1–24 (1989).

61. Angelsen, A. Agricultural expansion and deforestation: Modelling the impact of population, market forces and property rights. *Journal of Development Economics* **58**, 185–218 (1999).

62. Fearnside, P. M. Deforestation in Brazilian Amazonia: History, Rates, and Consequences. *Conservation Biology* **19**, 680–688 (2005).

63. Redo, D., Millington, A. C. & Hindery, D. Deforestation dynamics and policy changes in Bolivia’s post-neoliberal era. *Land Use Policy* **28**, 227–241 (2011).

64. Nepstad, D. *et al.* Inhibition of Amazon Deforestation and Fire by Parks and Indigenous Lands. *Conservation Biology* **20**, 65–73 (2006).

65. Ellis, E. A. & Porter-Bolland, L. Is community-based forest management more effective than protected areas? *Forest Ecology and Management* **256**, 1971–1983 (2008).

66. Bray, D. B. *et al.* Tropical Deforestation, Community Forests, and Protected Areas in the Maya Forest. *E&S* **13**, art56 (2008).

67. Porter-Bolland, L. *et al.* Community managed forests and forest protected areas: An assessment of their conservation effectiveness across the tropics. *Forest Ecology and Management* **268**, 6–17 (2012).

68. Duchelle, A. E. *et al.* Evaluating the opportunities and limitations to multiple use of Brazil nuts and timber in Western Amazonia. *Forest Ecology and Management* **268**, 39–48 (2012).

69. Deacon, R. T. Deforestation and the Rule of Law in a Cross-Section of Countries. *Land Economics* **70**, 414 (1994).

70. Deininger, K., Zegarra, E. & Lavadenz, I. Determinants and Impacts of Rural Land Market Activity: Evidence from Nicaragua. *World Development* **31**, 1385–1404 (2003).

71. Birdyshaw, E. & Ellis, C. Privatizing an open-access resource and environmental degradation. *Ecological Economics* **61**, 469–477 (2007).

72. Mendelsohn, R. & Balick, M. Private Property and Rainforest Conservation. *Conservation Biology* **9**, 1322–1323 (1995).

73. Gibson, C. C., McKean, M. A. & Ostrom, E. *People and Forests: Communities, Institutions, and Governance*. (MIT Press, 2000).

74. Baland, J.-M. & Platteau, J.-P. *Halting Degradation of Natural Resources - Is there a Role for Rural Communities?* (United Nations Food and Agriculture Organization and Oxford University Press, 2000).

75. Nolte, C., Agrawal, A., Silvius, K. M. & Soares-Filho, B. S. Governance regime and location influence avoided deforestation success of protected areas in the Brazilian Amazon. *Proceedings of the National Academy of Sciences* **110**, 4956–4961 (2013).

76. Ostrom, E. A General Framework for Analyzing Sustainability of Social-Ecological Systems. *Science* **325**, 419–422 (2009).

77. Naidu, S. C. Heterogeneity and Collective Management: Evidence from Common Forests in Himachal Pradesh, India. *World Development* **37**, 676–686 (2009).

78. Otsuka, K., Sakurai, T., Rayamajhi, S. & Pokharel, R. Efficiency of timber production in community and private forestry in Nepal. *Environment and Development Economics* **9**, 539–561 (2004).

79. Klingler, M. & Mack, P. Post-frontier governance up in smoke? Free-for-all frontier imaginations encourage illegal deforestation and appropriation of public lands in the Brazilian Amazon. *Journal of Land Use Science* **15**, 424–438 (2020).

80. Holden, S. & Yohannes, H. Land Redistribution, Tenure Insecurity, and Intensity of Production: A Study of Farm Households in Southern Ethiopia. *Land Economics* **78**, 573–590 (2002).

81. Deininger, K. & Jin, S. Tenure security and land-related investment: Evidence from Ethiopia. *European Economic Review* **50**, 1245–1277 (2006).

82. Fenske, J. Land tenure and investment incentives: Evidence from West Africa. *Journal of Development Economics* **95**, 137–156 (2011).

83. Robinson, B. E., Holland, M. B. & Naughton-Treves, L. Does secure land tenure save forests? A meta-analysis of the relationship between land tenure and tropical deforestation. *Global Environmental Change* **29**, 281–293 (2014).

84. Hargrave, J. & Kis-Katos, K. Economic Causes of Deforestation in the Brazilian Amazon: A Panel Data Analysis for the 2000s. *Environ Resource Econ* **54**, 471–494 (2013).

85. Arima, E. Y., Barreto, P., Araújo, E. & Soares-Filho, B. Public policies can reduce tropical deforestation: Lessons and challenges from Brazil. *Land Use Policy* **41**, 465–473 (2014).

86. Deacon, R. T. Deforestation and the Rule of Law in a Cross-Section of Countries. *Land Economics* **70**, 414–430 (1994).

87. FAO/SEAD. *Governança de terras: da teoria à realidade brasileira*. 378 (2017).

88. Leuzinger, M. & Lingard, K. The land rights of indigenous and traditional peoples in Brazil and Australia. *Revista de Direito Internacional* **13**,.

89. Soares-Pinheiro, P. Co-Management of Natural Resources in the Lowe Jurua Extractive Reserve, Central-West Brazilian Amazon. (University of Florida, 2018).

90. Carvalho, A. P. C. de & Carvalho, A. P. C. de. Tecnologias de governo, regularização de territórios quilombolas, conflitos e respostas estatais. *Horizontes Antropológicos* **22**, 131–157 (2016).

91. Sociedade Brasileira de Direito Público. *O Direito à terra das comunidades quilombolas*. http://www.sbdp.org.br/arquivos/material/432_Comunidades_quilombolas_direito_a_terra.pdf (2002).

92. Paixao, S. *et al.* Modeling indigenous tribes’ land rights with ISO 19152 LADM: A case from Brazil. *Land Use Policy* **49**, 587–597 (2015).
