# Supplementary material for: Land tenure drives Brazil’s deforestation rates across socio-environmental contexts
Source: Nat Commun. 2022 Oct 1;13:5759. doi: 10.1038/s41467-022-33398-3 (PMC9526711; doi:10.1038/s41467-022-33398-3)
Supplement: Supplementary file 2 — Reporting Summary [file 41467_2022_33398_MOESM2_ESM.pdf]

nature portfolio

Corresponding author(s): Andrea Pacheco

Last updated by author(s): September 11, 2022

Reporting Summary

Nature Portfolio wishes to improve the reproducibility of the work that we publish. This form provides structure for consistency and transparency in reporting. For further information on Nature Portfolio policies, see our Editorial Policies and the Editorial Policy Checklist.

Statistics

For all statistical analyses, confirm that the following items are present in the figure legend, table legend, main text, or Methods section.

n/a	Confirmed	
☐	☒	The exact sample size (*n*) for each experimental group/condition, given as a discrete number and unit of measurement
☒	☐	A statement on whether measurements were taken from distinct samples or whether the same sample was measured repeatedly
☐	☒	The statistical test(s) used AND whether they are one- or two-sided<br>*Only common tests should be described solely by name; describe more complex techniques in the Methods section.*
☐	☒	A description of all covariates tested
☐	☒	A description of any assumptions or corrections, such as tests of normality and adjustment for multiple comparisons
☐	☒	A full description of the statistical parameters including central tendency (e.g. means) or other basic estimates (e.g. regression coefficient) AND variation (e.g. standard deviation) or associated estimates of uncertainty (e.g. confidence intervals)
☐	☒	For null hypothesis testing, the test statistic (e.g. *F*, *t*, *r*) with confidence intervals, effect sizes, degrees of freedom and *P* value noted<br>*Give P values as exact values whenever suitable.*
☒	☐	For Bayesian analysis, information on the choice of priors and Markov chain Monte Carlo settings
☒	☐	For hierarchical and complex designs, identification of the appropriate level for tests and full reporting of outcomes
☒	☐	Estimates of effect sizes (e.g. Cohen's *d*, Pearson's *r*), indicating how they were calculated

Our web collection on statistics for biologists contains articles on many of the points above.

Software and code

Policy information about availability of computer code

Data collection	All data used in this study was publicly available, downloaded and processed using the steps outlined at https://github.com/pacheco-andrea/tenure-defore-br
Data analysis	All the code used to analyze the data in this study is publicly available and uses R (versions 3.5.1-4.0.2), and the specific R packages used are listed at each step they are needed at https://github.com/pacheco-andrea/tenure-defore-br. This includes, namely: cem, generalize, margins, and rbounds.

For manuscripts utilizing custom algorithms or software that are central to the research but not yet described in published literature, software must be made available to editors and reviewers. We strongly encourage code deposition in a community repository (e.g. GitHub). See the Nature Portfolio guidelines for submitting code & software for further information.

Data

Policy information about availability of data

All manuscripts must include a data availability statement. This statement should provide the following information, where applicable:

- Accession codes, unique identifiers, or web links for publicly available datasets
- A description of any restrictions on data availability
- For clinical datasets or third party data, please ensure that the statement adheres to our policy

All data used in the figures and empirical analyses of this study are publicly available (see refs. 17, 18, 43-45). Processed data from these sources are available as Supplementary Data 1, and full regression outputs and Rosenbaum bounds are available as Supplementary Data 2-3. All supplementary data are accessible at (https://doi.org/10.5281/zenodo.7068678) (see ref. 52). The data generated in this study on the estimation of effects per each spatiotemporal scale are provided in the Source Data file.

Field-specific reporting

Please select the one below that is the best fit for your research. If you are not sure, read the appropriate sections before making your selection.

☐ Life sciences ☒ Behavioural & social sciences ☐ Ecological, evolutionary & environmental sciences

For a reference copy of the document with all sections, see nature.com/documents/nr-reporting-summary-flat.pdf

Behavioural & social sciences study design

All studies must disclose on these points even when the disclosure is negative.

Study description	This study uses quasi-experimental study design, specifically, matching, weighing of observations, and regression analysis (generalized linear models) to draw causal inferences from observational data.
Research sample	The study sample of this study is the entire population of mapped land parcels in Brazil, which is specifically subset using statistical matching.<br>This is a compilation of land tenure data by Imaflora: Atlas - The Geography of Brazilian Agriculture (v. 1812). This data compilation gathers and harmonizes data from 18 official sources. More information on this data compilation can be found at: Imaflora, GeoLab (ESALQ/USP), Royal Institute of Technology in Stockholm (KHT) & Instituto Federal de Educação, Ciência e Tecnologia de São Paulo (IF/SP). Atlas - The geography of Brazilian agriculture. (2018).<br>The atlas can be found at: http://atlasagropecuario.imaflora.org/mapa
Sampling strategy	Because we use observational data, we did not use a traditional sampling procedure in this study. Instead, we included the entire population of mapped land tenure parcels in Brazil in our analyses. However, we use a quasi-experimental design (statistical matching) to address the bias that would arise due to treatment assignment not being independent of the outcome. Therefore, sample sizes were determined by the number of attainable "matches" of "treatment" and "control" observations. We proceed with our analysis of causal effects in cases where the matching procedure yielded sufficiently large data subsets for unbiased parameter estimation. For most tenure-regime comparisons and spatiotemporal scales this meant a sample size of 44 to 34,218 of unique observations of land parcels. This corresponds to ≥6 observations per parameter, which is recommended by Vittinghoff & McCulloch (2007).
Data collection	We did not collect data for this study. Instead, we use a compilation of land tenure data by Imaflora: Atlas - The Geography of Brazilian Agriculture (v. 1812). This data compilation gathers and harmonizes data from 18 official sources. More information on this data compilation can be found at: Imaflora, GeoLab (ESALQ/USP), Royal Institute of Technology in Stockholm (KHT) & Instituto Federal de Educação, Ciência e Tecnologia de São Paulo (IF/SP). Atlas - The geography of Brazilian agriculture. (2018).<br>The atlas can be found at: http://atlasagropecuario.imaflora.org/mapa
Timing	As stated above, we did not collect data for this study. Instead, we use a compilation of land tenure data which do not have an associated date of collection or creation. However, we associate these data with annual land-cover data in Brazil, which are based on remote sensing and vegetation mapping products. Therefore, we use land-cover data produced annually from 1985-2018.
Data exclusions	We pre-established the exclusion of military lands, urban and transport-related lands, and water from our analyses, as these categories were less relevant to the hypothesized mechanisms relating land-tenure regimes to forest-to-agriculture conversion. We also pre-established the exclusion of properties belonging to the Terra Legal program as this program only started in 2009 and thus these properties experienced shifts in tenure categories during our study period which could bias our results. Moreover, the outcomes of this program were the focus of a different study (Probst et al., 2020, Nat. Sustain)
Non-participation	Non-participation in an experiment/survey is not relevant in this study because we use observational data.
Randomization	Observations were grouped into quasi-treatment and quasi-control groups using coarsened-exact matching based on a set of covariates known to influence forest-to-agriculture conversion which have been shown o be relevant for policy-makers when deciding on shifts in tenure regimes under many different contexts. These include market accessibility, agricultural suitability, human population density, and parcel area.

Reporting for specific materials, systems and methods

We require information from authors about some types of materials, experimental systems and methods used in many studies. Here, indicate whether each material, system or method listed is relevant to your study. If you are not sure if a list item applies to your research, read the appropriate section before selecting a response.

Materials & experimental systems

n/a	Involved in the study
☒	☐ Antibodies
☒	☐ Eukaryotic cell lines
☒	☐ Palaeontology and archaeology
☒	☐ Animals and other organisms
☒	☐ Human research participants
☒	☐ Clinical data
☒	☐ Dual use research of concern

Methods

n/a	Involved in the study
☒	☐ ChIP-seq
☒	☐ Flow cytometry
☒	☐ MRI-based neuroimaging
